# Small-molecule inhibition of STAT3 in radioresistant head and neck squamous cell carcinoma

**DOI:** 10.18632/oncotarget.8368

**Published:** 2016-03-25

**Authors:** Uddalak Bharadwaj, T. Kris Eckols, Xuejun Xu, Moses M. Kasembeli, Yunyun Chen, Makoto Adachi, Yongcheng Song, Qianxing Mo, Stephen Y. Lai, David J. Tweardy

**Affiliations:** ^1^ Department of Infectious Disease, Infection Control and Employee Health, The University of Texas MD Anderson Cancer Center, Houston, Texas, USA; ^2^ The Key Laboratory of Natural Medicine and Immuno-Engineering, Henan University, Kaifeng, China; ^3^ Department of Head and Neck Surgery, Division of Surgery, The University of Texas MD Anderson Cancer Center, Houston, Texas, USA; ^4^ Department of Pharmacology, Baylor College of Medicine, Houston, Texas, USA; ^5^ Department of Medicine, Division of Biostatistics, Dan L. Duncan Cancer Center, Section of Hematology/Oncology, Baylor College of Medicine, Houston, Texas, USA; ^6^ Department of Molecular & Cellular Oncology, The University of Texas MD Anderson Cancer Center, Houston, Texas, USA

**Keywords:** STAT3, HNSCC, C188-9, cancer, small molecule

## Abstract

While STAT3 has been validated as a target for treatment of many cancers, including head and neck squamous cell carcinoma (HNSCC), a STAT3 inhibitor is yet to enter the clinic. We used the scaffold of C188, a small-molecule STAT3 inhibitor previously identified by us, in a hit-to-lead program to identify C188-9. C188-9 binds to STAT3 with high affinity and represents a substantial improvement over C188 in its ability to inhibit STAT3 binding to its pY-peptide ligand, to inhibit cytokine-stimulated pSTAT3, to reduce constitutive pSTAT3 activity in multiple HNSCC cell lines, and to inhibit anchorage dependent and independent growth of these cells. In addition, treatment of nude mice bearing xenografts of UM-SCC-17B, a radioresistant HNSCC line, with C188-9, but not C188, prevented tumor xenograft growth. C188-9 treatment modulated many STAT3-regulated genes involved in oncogenesis and radioresistance, as well as radioresistance genes regulated by STAT1, due to its potent activity against STAT1, in addition to STAT3. C188-9 was well tolerated in mice, showed good oral bioavailability, and was concentrated in tumors. Thus, C188-9, either alone or in combination with radiotherapy, has potential for use in treating HNSCC tumors that demonstrate increased STAT3 and/or STAT1 activation.

## INTRODUCTION

Signal transducer and activator of transcription 3 (STAT3) is a member of a family of seven closely related proteins responsible for transmission of peptide hormone signals from the extracellular surface of cells to the nucleus [1]. STAT3 is a master regulator of several key hallmarks and enablers of cancer [2] including cell proliferation, resistance to apoptosis, metastasis, immune evasion, tumor angiogenesis, epithelial mesenchymal transition (EMT), response to DNA damage, and the Warburg effect [3-6]. STAT3 also is a key mediator of oncogene addiction [7] and supports the self-renewal of tumor-initiating cancer stem cells that contribute to cancer initiation, cancer maintenance, and relapse [8, 9] in several types of tumors. STAT3 activity is increased in ~50% of all cancers [10], due, in many instances, to activation of signaling molecules upstream of STAT3, including receptor tyrosine kinases (RTK; e.g. epidermal growth factor receptor, EGFR), tyrosine kinase-associated receptors (e.g. the family of IL-6 cytokine receptors or G-protein coupled receptors, GPCR) [11, 12], and Src kinases (e.g. Src, Lck, Hck, Lyn, Fyn, or Fgr) [12, 13]. Thus, STAT3 is an attractive target for drug development to treat many types of cancer including head and neck squamous cell carcinoma (HNSCC).

HNSCC was the first human cancer demonstrated to depend on constitutively activated STAT3 for growth [3, 14]. Inhibition of the EGFR or TGFα depletion in HNSCC resulted in growth inhibition and diminished STAT3 DNA-binding activity [14]. Moreover, direct inhibition of STAT3 resulted in growth inhibition of HNSCC cell lines [14, 15]. The reported incidence of activated EGFR in HNSCC tumors and cell lines varies from 5% to 90% [16, 17], however, suggesting that STAT3 activation in HNSCC may occur independently of EGFR activation. Specific inhibition of EGFR failed to prevent STAT3 activation in some HNSCC cell lines; rather, an autocrine/paracrine IL-6/gp130 loop was demonstrated in these cell lines [18]. In addition to its contribution to HNSCC oncogenesis, there is increasing evidence suggesting a role for STAT3 and, more recently, STAT1 in resistance of HNSCC tumors to ionizing radiation (IR) [19-23].

STAT3 has been targeted in HNSCC xenograft models using the small-molecule, STAT3 inhibitor, Stattic, which was shown to enhance IR-sensitivity [19, 24]. However, it has a low maximum tolerated dose in mice due, most likely, to off-target effects mediated by its covalent mechanism of action; consequently, the pathway for Stattic to enter the clinic is uncertain. EGFR inhibitors–cetuximab, gefitinib or erlotinib–combined with IR with or without chemotherapy showed encouraging results [25]; however, well-defined markers for either patient selection or prediction of prognosis did not emerge from these studies. Thus, there remains a need for a STAT3 inhibitor suitable for clinical use either alone or in combination with IR or chemotherapy to improve treatment outcomes in HNSCC.

Ligand-engagement of receptors for growth factors or cytokines [11, 26] causes receptor oligomerization and activation of intrinsic or receptor-associated tyrosine kinases, respectively. These activated kinases phosphorylate receptor tyrosine residues creating docking sites for recruitment of cytoplasmic STAT3 [11, 26]. STAT3 docks to receptor phosphotyrosyl (pY) peptide sites through its Src-homology (SH) 2 domain, which leads to its phosphorylation on Y^705^ followed by STAT3 tail-to-tail homodimerization (SH2 domain of each monomer binds the pY^705^ peptide domain of its partner). STAT3 homodimers accumulate in the nucleus, where they bind to specific STAT3 response elements in the promoter of target genes and regulate their transcription. Several small molecule drug-development programs have emerged directed at identifying drug-like compounds that target STAT3 at one or more stages of its activation [27-29].

Using virtual ligand screening, we docked 920,000 small molecules from 8 chemical libraries into the pY-binding pocket of the STAT3 SH2 domain and identified 3 hits–C3, C30, and C188–as direct STAT3 inhibitors. C188 demonstrated the greatest activity of the three [9, 29, 30]. Using C188 as the scaffold, we performed 2D similarity screening and 3D pharmacophore analysis and identified C188-9, which in the studies outlined herein proved to be more potent in all assays for STAT3 inhibitory activity tested including inhibition of growth of HNSCC xenografts. C188-9 also has a high maximum tolerated dose, is orally bioavailable, and has great potential for clinical use either alone or in combination with IR or chemotherapy for the treatment of HNSCC.

## RESULTS

### 2-D fingerprint screening followed by 3-D pharmacophore sorting identified potent second generation STAT3 probes

We previously [29] performed virtual ligand screening (VLS) of 920,000 compounds and identified three compounds C3, C30 and C188 with promising reversible inhibitory activities [29] in a STAT3 (pY)-peptide ligand binding assay and in a ligand-induced STAT3 phosphorylation assay. C188, in particular, was highly active in inducing apoptosis of the breast cancer cell line MB-MDA-468 *in vitro* (EC_50_= 0.7 μM). To identify more potent STAT3 probes, we used the scaffold of C188 as a reference structure (Table 1) and performed 2-D fingerprint screening of 490,000 compounds in a Life Chemicals compound database. We used 100 percent similarity as cutoff and employed the Tanimoto coefficient as the similarity method (Unity/Sybyl/Tripos) [31], which identified 207 compounds. The molecular features of these compounds were combined and the 3D pharmacophore structures were compared using a matrix that incorporated the structural features as distance bins. Compounds were ranked in decreasing order of pharmacophore similarity and the top 39 compounds with pharmacophore scores greater than 70 were purchased for further testing in our assays for STAT3 inhibition (Supplementary Table S1). Of the first twenty, nine compounds (C188-1, C188-7, C188-8, C188-9, C188-15, C188-16, C188-17, C188-18, C188-19) showed improved inhibition of STAT3 binding to pY-peptide (Table 1 and Supplementary Table S1, representative binding curves shown in Figure 2A), while two compounds (C188-10 and C188-14) had activity similar to C188. The remaining nine compounds had activity less than C188. None of compounds that ranked below 21 showed potency in inhibiting STAT3 binding to pY-peptide that was similar or improved over C188.

**Table 1:** Summary of features, activities, MTDs, and tumor PK of C188 and C188-9

Features	C188	C188-9
***Chemical Features***		
Structure	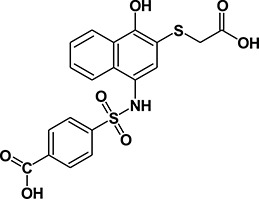	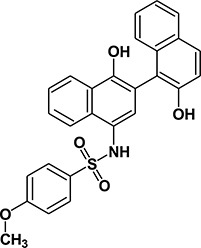
Log P	-	5.2
Solubility (μM)^1^	-	13.1
***Inhibitory Activity*** (Mean ± SEM, n ≥ 2)		
**STAT3-pY peptide binding** (SPR)	7.5 ± 3.5	2.5 ± 2.1
Ki	37.3 nM	12.4 nM
**Ligand stimulated phosphorylation** (Phosphoflow)		
pSTAT3 (G-CSF)	16.8 ± 20.1	8.9 ± 5.8
pSTAT1 (IFNγ)	15.0 ± 0.0	9.5 ± 5.6
**G-CSF Induced Phosphorylation** (Luminex)		
pSTAT3	16.2 ± 2.3	3.7 ± 1.9
pSTAT1	18.6 ± 4.7	4.1 ± 3.3
**Constitutive pSTAT HNSCC cells**		
**UM-SCC-17B**		
pSTAT3	15.4 ± 9.2	10.6 ± 0.7
**Anchorage dependent growth** (MTT)		
UM-SCC-17B	6.3 ± 0.8	3.2 ± 0.6
***MTD*** *(mg/Kg/day)*		
Mice: 14 days	100	100
***Plasma PK***: *IP and oral (10 mg/Kg)*		
Area under the curve (AUC, IP/oral, μg-hr/mL)	ND	12.5/12.5
***Tumor PK:** IP (10 mg/Kg; mouse)*		
Tumor level/plasma level (mg/ml [μM])^2^	ND	5.0 [10.4]/ 1.9 [4.0]
Tumor: Plasma ratio	ND	2.6

ND: Not Done; Cells used: G-CSF induced pSTAT3 and IFN-γ induced pSTAT1 by Phosphoflow: Kasumi-1, G-CSF induced pSTAT/GAPDH by Luminex (Kasumi1),

1 60 min at 37°C in PBS, pH 7.4,

2 Tumor and plasma harvested 1 hr following IP dose.

**Figure 1:**
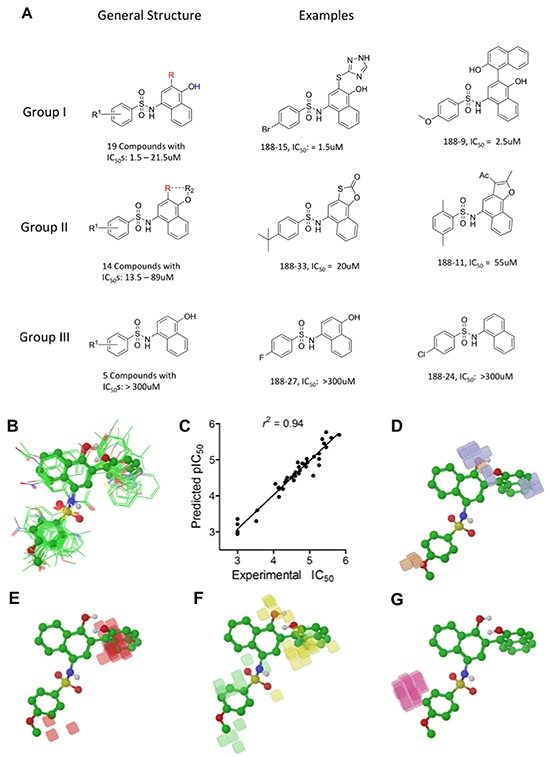
Structure Activity Relationship (SAR) of C188 and similar compounds **A.** SAR grouping of 37 C188-like STAT3 probes. Thirty-seven of the 39 C188-like compounds can be divided into three structural groups with activity ranging from highest to lowest. The most potent Group I compounds contain a variety of groups, such as a triazole-3-yl-mercapto (188-15) or a hydroxynaphthalene (188-9), at the 3-position of the naphthylamine ring (the -R group highlighted in red). Group II compounds with intermediate potency contain a 5-membered ring that combines the 3-R and 4-OR^2^ groups, such as a furan (188-11). The least potent Group III probes do not contain a substitution at the 3-position. **B–G.** Quantitative SAR of compounds. Alignment of C188 and C188-1 through C188-39 showing (B) only heavy atoms and polar hydrogens displayed for clarity, with C188-9 in ball and stick model; **C.** Correlation between experimental and predicted pIC_50_ values; **D.** Phase H-bond donor fields, superimposed with the aligned structure of C188-9, blue favorable, orange disfavored; **E.** hydrophobic fields, red favorable; **F.** electron-withdrawing fields, yellow favorable, light green disfavored; **G.** negative ionic fields, pink favorable.

We performed SAR analysis using C188 and its 39 derivatives to gain insight into structural features critical for binding of C188 and its derivatives to STAT3. All 39 C188-like compounds, including C188 itself, are derivatives of N-naphth-1-yl benzenesulfamide. SAR revealed that 37 of the 39 C188-like compounds could be divided into three structural groups (I, II and III) with decreasing activity (Figure 1A). Addition of a variety of groups (the -R group highlighted in red in the general structure of Group I in Figure 1A), such as a triazole-3-yl-mercapto (C188-15) or a binapthalyl group (C188-9), to the 3-position of the naphthylamine ring yields the Group I compounds, which are the most potent group of STAT3 probes. The –R group appears to be the most important contributor to the inhibitory activity of group I probes: a total of eight 3-substituents are found in Group I compounds, which enhanced the activity by one or more orders of magnitude. Most STAT3 probes in Group II contain a 5-membered ring that combines the 3-R and 4-OR_2_ groups, such as a furan (C188-11). However, the compounds in this group are, on an average, ~5x less active than the Group I compounds, which suggests the H atom of the 4-hydroxy group (highlighted in blue in the general structure of Group I in Figure 1A) is important, e.g., involved in a favorable H-bond with the protein. Lack of ability to form the H-bond might attribute to the weaker activities of Group II probes.

To understand the SAR of these compounds in a more quantitative and predictive manner, we performed a 3-D quantitative structure activity relationship (QSAR) study, using the program Phase in Schrödinger (version 2010). These 40 compounds were built and their energy and geometry minimized using the OPLS-2005 force field in Maestro (version 9.1 in Schrödinger. They were then aligned using the “flexible ligand alignment” module in Maestro, which recognizes common features within these molecules (e.g., similar partial charge, hydrophobicity, aromaticity and H-bond donor/acceptor). The aligned ligands (Figure 1B) were imported into the Phase program and a partial least squares (PLS) method was applied to correlate the STAT3 inhibitory activities (pIC_50_, or -log_10_IC_50_) of these compounds with the Phase field data calculated based on their aligned 3-D structures (Figure 1C). As shown in Supplementary Table S2, the QSAR model (training set) yielded *r*^2^ = 0.94 (Figure 1C), *q*^2^ (no. of factors) = 0.58 (4), *F*-test = 133.0, and a pIC_50_ error of 0.19. To further validate the model, five leave-5-out training test sets were performed with good results yielding an *r*^2^ ≥ 0.90, *q*^2^ ≥ 0.57, *F*-test ≥ 95.8, as well as pIC_50_ errors of ≤ 0.24. 3-D QSAR was used to visualize the 4 major factors contributing to activity: H-bond donor (Figure 1D, blue favorable, orange disfavored), hydrophobic fields (Figure 1E, red favorable), electron-withdrawing fields (Figure 1F, yellow favorable, light green disfavored) and negative-ionic Phase fields (Figure 1G, pink favorable).

### C188-9 emerged as the lead STAT3 probe and binds to STAT3 with high affinity

**Figure 2:**
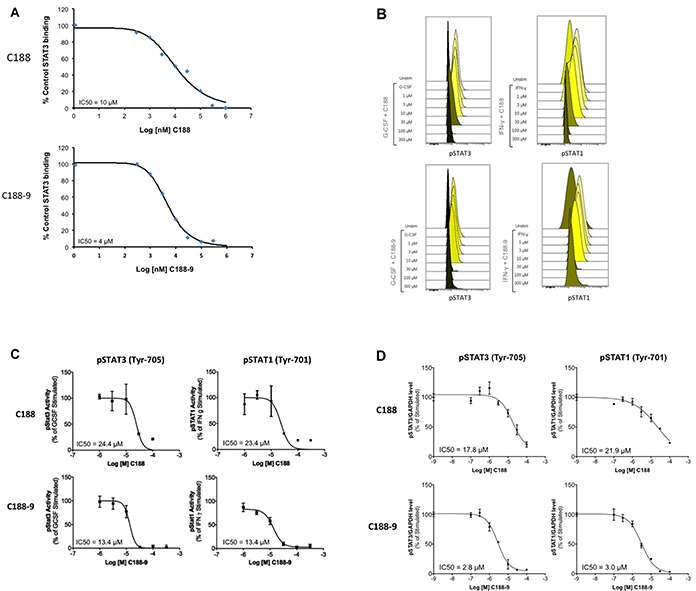
Inhibition of STAT3 activities by C188 and C188-9 **A.** Inhibition of recombinant STAT3 (200nM) binding to Biacore sensor-chip-immobilized phosphododecapeptide ligand (12 amino acids surrounding and including pY1068 within EGFR) by C188 (0.1 to 1000 μM, top) and C188-9 (0.1 to 1000 μM, bottom) by SPR. The equilibrium binding levels obtained ± compound were normalized (resonance obtained in the presence of compound ÷ the resonance obtained in the absence of compound × 100) and plotted against Log [nM] C188 (or C188-9) and IC_50_ calculated (value shown in inlay). **B.** Inhibition of ligand-stimulated STAT phosphorylation, measured by phosphoflow. Serum-starved (1 hour) Kasumi-1 cells, pre-incubated with compound/DMSO (1 hour), were treated with G-CSF (10 ng/ml, 15 min, left two panels) or IFN-γ (10ng/ml, 15 min, right panel). Cells were permeabilized and stained with Alexa647-pSTAT3, and PE-pSTAT1 antibodies and analyzed on BD LSR2. FCS files were uploaded to Cytobank for pSTAT3 and pSTAT1 quantitation. Histograms depicting pSTAT3 and pSTAT1 levels are shown. **C.** Mean fluorescence (pSTAT3/1) levels were plotted as function the Log [M] compound, and IC_50_ calculated using GraphPad. 1D shows IC_50_ curve from representative experiments. **D.** Inhibition of ligand-stimulated STAT phosphorylation, measured by Luminex. Serum-starved (1 hour) Kasumi-1 cells, pre-incubated with compound/DMSO (0/0.1/0.3/1/3/10/100 μM, 1 hour), were treated with G-CSF (10 ng/ml, 15′). Total protein extracts of cells were assayed for pSTAT3, pSTAT1, and GAPDH levels by Luminex. GAPDH-normalized pSTAT3 or pSTAT1 values were divided by this ratio for untreated cells and expressed in percentage. These values were plotted as a function of Log [M] compound, and IC_50_ values calculated using GraphPad. Upper panel shows data from representative experiments with C188 and lower panel shows those with C188-9.

**Figure 3:**
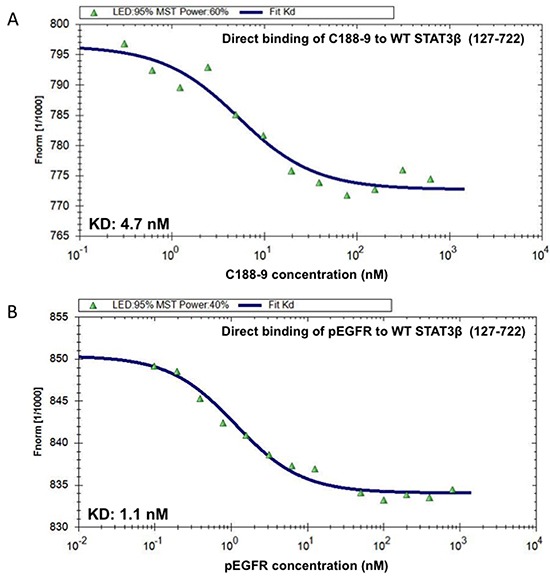
C188-9 binds to STAT3 with high affinity Increasing concentrations of C188-9 (0.305 to 10,000 nM; panel **A.**) and the phosphotyrosyl (pY)-dodecapeptide based on the portion of the EGFR surrounding Y1068 (EGFR pY-1068; 0.025 to 800 nM; panel **B.**) were incubated with a constant concentration (80 nM) of fluorescently labeled STAT3 (aa residues 127-722). Fluorescence was measured continuously before and after application of an infrared laser. The change in fluorescence (F_norm_) was calculated from the ratio of fluorescence immediately before heating and 30 seconds after heating and plotted against the logarithm of the different concentrations of C188-9 or EGFR pY-1068 (A and B); the sigmoidal binding curve was used to calculate the dissociation constant K_D_.

Phosphoflow analysis of the C188 and derivatives, which examined the ability of each to inhibit G-CSF-induced STAT3 phosphorylation (Supplementary Table S1), revealed that four compounds (C188-7, C188-8, C188-9 and C188-15) inhibited G-CSF-induced pSTAT3 levels with greater potency (IC_50_ = 3.3 – 10.5 μM) than C188 (IC_50_=16.8 μM; Table 1, Figure 2B, 2C). In studies not shown, C188-7, C188-8, and C188-15 inhibited normal murine bone marrow colony formation, while C188-9 did not (Tweardy et al 2010, unpublished). Assessment of the ability of C188-9 to inhibit G-CSF-induced pSTAT3 using a Luminex bead-based assay (Table 1, Figure 2D) also revealed improvement in STAT3 inhibitory activity of C188-9 (IC_50_=3.7 μM) compared to C188 (IC_50_=16.2 μM, respectively). C188-9 also was found to bind to STAT3 with high affinity (K_D_=4.7±0.4 nM), as determined by microscale thermophoresis (MST; Figure 3A). The affinity of binding of EGFR pY1068-peptide to STAT3 determined by MST was K_D_=1.1±0.1 nM (Figure 3B). Using the Cheng-Prussof equation [K_i_=SPR IC_50_/(1+[STAT3]/K_D_] [32], the K_i_ for C188-9 is calculated to be 12.4 nM (Table 1), where the C188-9 SPR IC_50_=2,500 nM, [STAT3]=200 nM, and the K_D_ of EGFR pY-1068 binding to STAT3 binding to STAT3 is 1.1 nM. Thus, there is a close match between the K_D_ of C188-9 measured by MST and its calculated Ki.

### Comparison of C188-9 vs. C188 in targeting STAT3 in HNSCC

**Figure 4:**
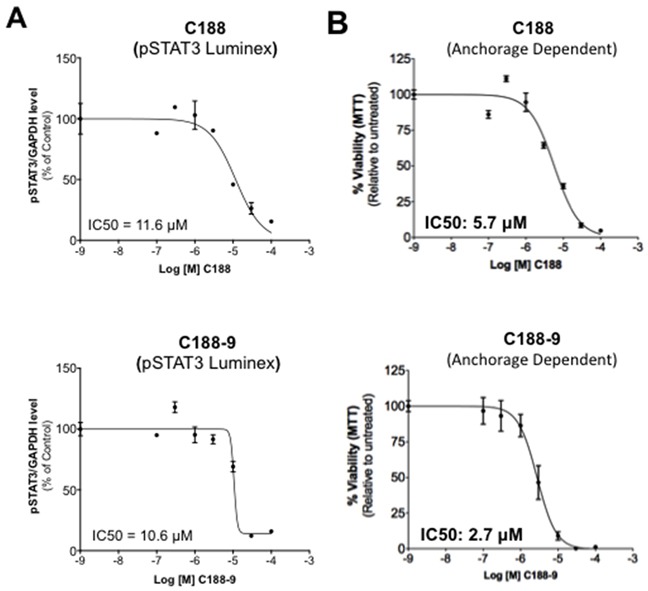

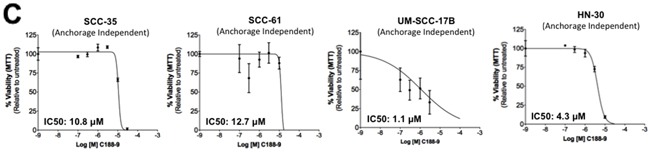
Inhibition of constitutive pSTAT3 and pSTAT1 and resultantly growth of HNSCC cells by C188-9 **A.** Lysates from asynchronous cultures of UM-SCC-17B cells treated with DMSO or C188 or C188-9 with DMSO or increasing doses (0/0.1/0.3/1/3/10/100 μM) of C188 or C188-9 for 24 hrs, assayed for pSTAT3/pSTAT1 and GAPDH by Luminex. GAPDH-normalized pSTAT3 or pSTAT1 values were divided by that for untreated cells and expressed in percentage. These values were plotted as a function of Log [M] compound, and IC_50_ values calculated using GraphPad. Upper panel shows data from representative experiments with C188 and lower panel shows those with C188-9. **B.** Effect of C188 and C188-9 on anchorage dependent growth of UM-SCC-17B cells. Cells were cultured for 48 hrs in complete DMEM with 10% FBS ± C188 0r C188-9 (0/0.1/0.3/1/3/10/100 μM) in cell-culture-treated 96-well plates. Viable cells quantitated using MTT. Relative % viability was measured by (viability after any treatment ÷ viability of untreated cells × 100) and plotted as a function of Log [M] C188/C188-9, and IC_50_ values calculated using GraphPad. Data show representative experiments from ≥ 2 replicates. **C.** SCC-35, SCC-61, UM-SCC-17B and HN30 cells were treated with increasing doses of C188-9 for 72 hrs and IC50 for ability of C188-9 to inhibit anchorage independent growth were calculated as in (B). Representative curves are shown. Mean IC50 values are shown in Table 2.

We compared the abilities of C188-9 and C188 to target STAT3 in the HNSCC cell line, UM-SCC-17B, previously shown to have constitutively activated STAT3 [19]. UM-SCC-17B cells were incubated with various doses (0/0.1/0.3/1/3/10/30μM) of C188 or C188-9 for 24 hrs. C188-9 (IC_50_=10.6 ± 0.7 μM, Table 1, Figure 4A), was more potent in reducing constitutive pSTAT3 levels at 24 hrs compared to C188 (IC_50_=15.4 ± 9.2 μM, Table 1, Figure 4A). C188-9 also was more potent than C188 in inhibiting and anchorage-dependent growth (IC_50_ 3.2 μM vs 6.3 μM, Table 1, Figure 4B).

**Table 2:** Inhibition of constitutive pSTAT3/1 and anchorage independent growth of HNSCC cell lines by C188-9

HNSCC Cell line	Constitutive pSTAT3	Constitutive pSTAT1	IC_50_ pSTAT3	IC_50_ pSTAT1	IC_50_ Cell growth (MTT)
SCC-35	++	-	22.8 ± 6.3	NA	10.8 ± 0.0
SCC-61	++	+	21.5 ± 7.1	28.5 ± 0.9	14.8 ± 2,9
UM-SCC-17B	++	++	10.6 ± 0.7	19.1 ± 15.5	0.7 ± 0.6
HN30	+/−	+++	21.5 ± 8.3	5.0 ± 6.0	4.4 ± 0.1

Relative levels of pSTAT3 and pSTAT1 were indicated with negative and positive signs based on Luminex data shown in Supplementary Figure S1 based on their relative levels and whether these levels were above or below the levels in non-tumor line HEEpiC. Lysates from asynchronous cultures of SCC-35, SCC-61 and HN30 cells treated with DMSO or increasing doses (0/0.1/0.3/1/3/10/100 μM) of C188-9 for 36 hrs, assayed for pSTAT3/pSTAT1 and GAPDH by Luminex. GAPDH-normalized pSTAT3 or pSTAT1 values were divided by that for untreated cells and expressed in percentage. These values were plotted as a function of Log [M] compound, and IC_50_ values calculated using GraphPad. IC50s for inhibition of anchorage independent growth (Cell growth) was measured as described in methods and Legends of Figure 4C.

To examine whether C188-9 targeted STAT3 in other HNSCC cells, we used Luminex assays to determine the levels of constitutively phosphorylated STAT3 in 10 HNSCC cell lines (SCC-9, SCC-15, HN5, UM-SCC-1, SCC-61, SQ-20B, SCC-35, UM-SCC-17B, HN30 and HN31), as well as in the primary human esophageal epithelial cell line (HEEpiC). Seven of 10 cell lines had increased basal pSTAT3 levels (Supplementary Figure S1A). We examined the effect of C188-9 in reducing pSTAT3 levels in four cell lines–SCC-35, SCC-61 and UM-SCC-17B with the highest pSTAT levels and HN30 with only slightly elevated pSTAT3 levels (Supplementary Figure S1A). C188-9 reduced constitutive pSTAT3 levels in all four cell lines (IC_50_ ranging from 10.5 to 22.8 μM, Table 2). In addition, C188-9 inhibited anchorage independent growth of all the four cell lines (IC_50_ ranging from 0.7 to 14.8 μM, Table 2, Figure 4C).

**Figure 5:**
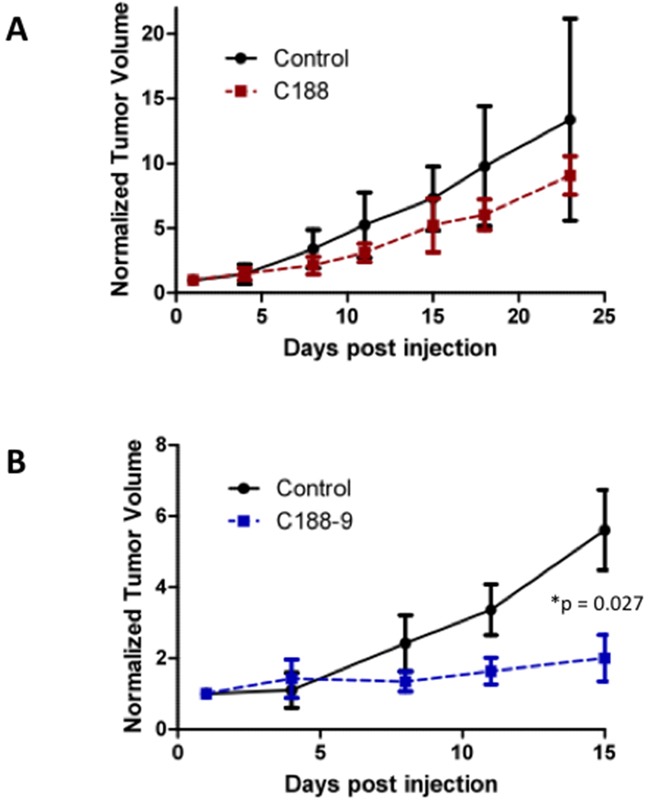

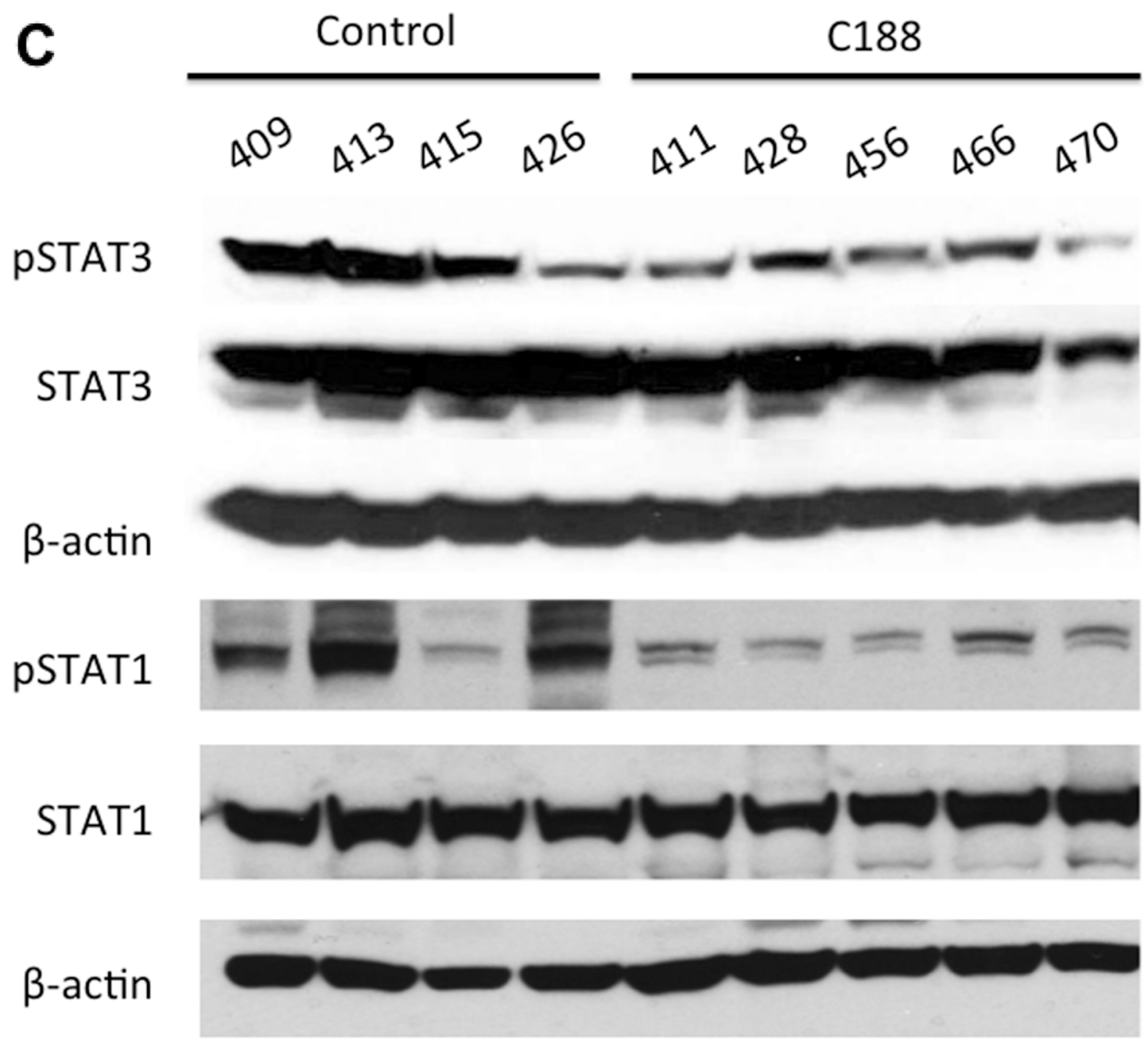

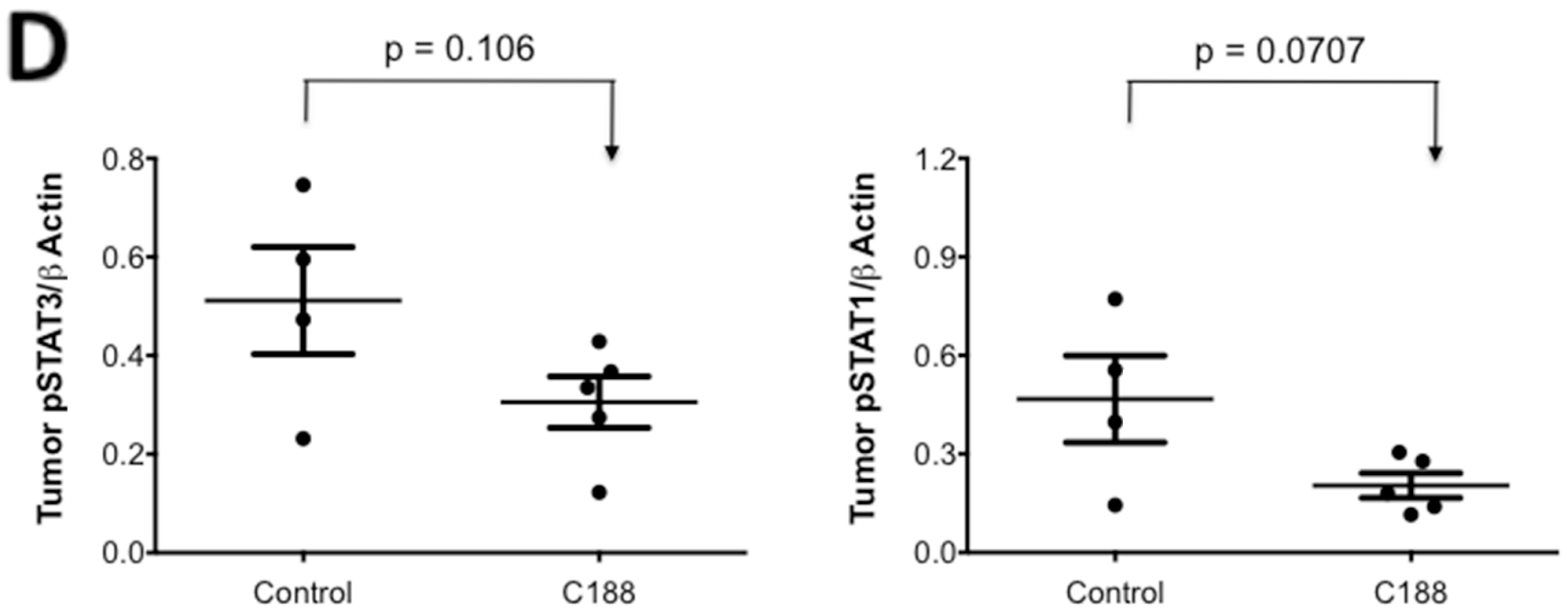

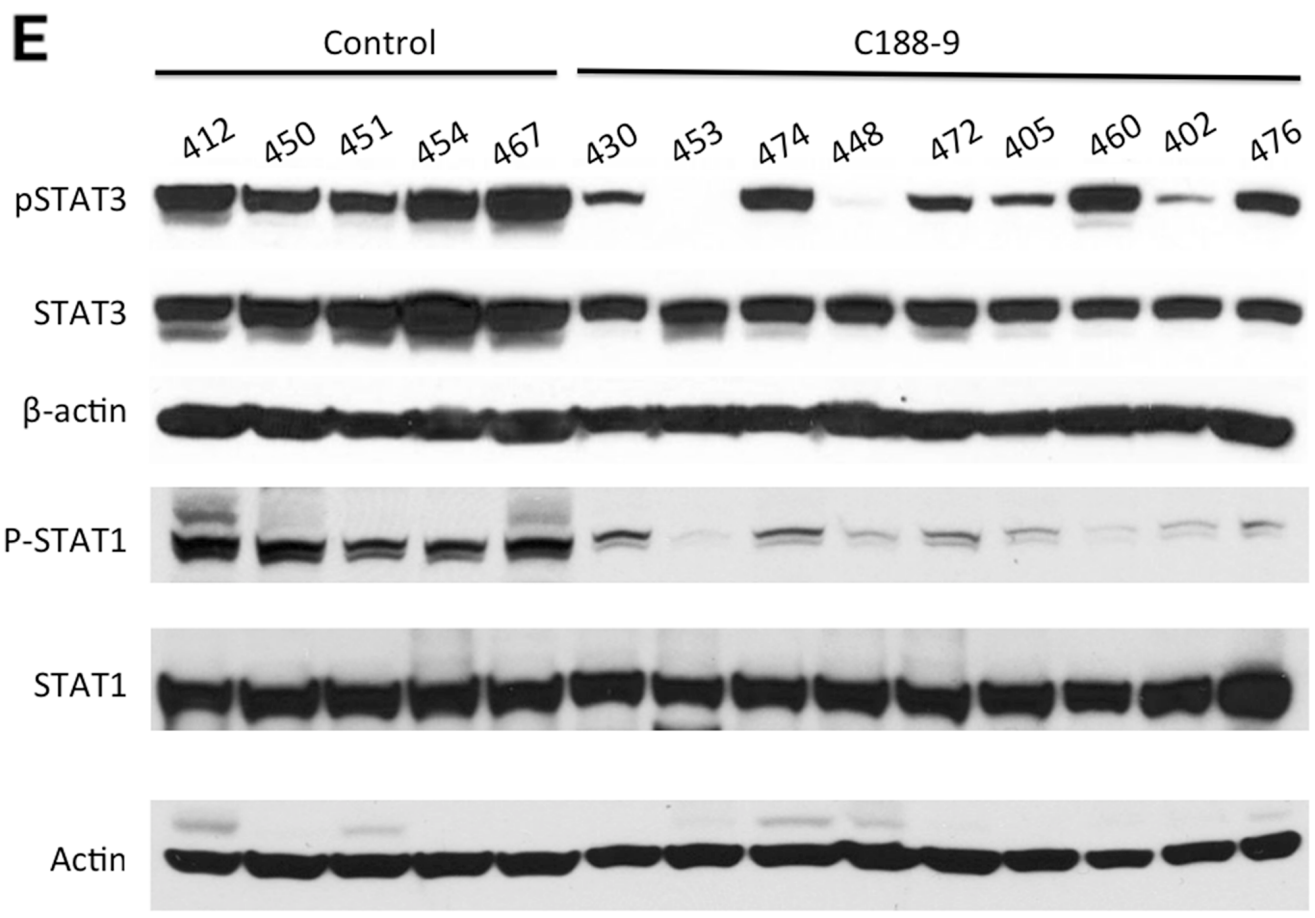

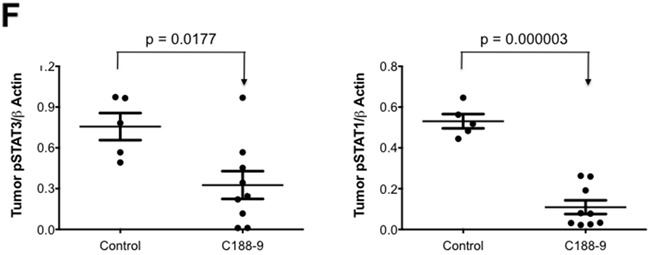
C188-9 efficiently targets STAT3 in HNSCC xenografts and inhibits tumor growth in nude mice UM-SCC-17B cells (1.5 × 10^6^) were injected into the tongues of athymic, 8–10 week old, male, nude mice (NCI, Frederick, MD, USA). Once tumors were established, mice (5/group) were randomized (average tumor vol ~ 15-20 mm^3^) to receive 5 times a week, intraperitoneal injections of either DMSO or C188 (50 mg/Kg) or C188-9 (100 mg/Kg). Tumor volumes were measured twice weekly. Average tumor volumes (0.5 × (long dimension) × (short dimension)^2^ were calculated and normalized to the volume at first day of treatment and plotted along the Y axis, for C188 **A.** and C188-9 **B.** treatments. Comparison was done by t test (* p<0.05). After injections, mice were euthanized, and lysates of tumors from C188-treated **C.** or C188-9-treated **E.** mice were immunoblotted for pSTAT3, total STAT3, β-actin, pSTAT1, total STAT1. Whisker plots of β-actin-normalized pSTAT3 (left panels) and pSTAT1 values (right panels) for C188 treatment **F.** are shown with the differences in means compared using t test.

We next examined the effect of C188 and C188-9 on growth of tumor xenografts, which revealed that the greater growth inhibitory activity of C188-9 vs. C188 extended to UM-SCC-17B cell line xenografts. While established UM-SCC-17B xenograft tumors continued to grow in nude mice that received C188 (50 mg/kg/day; Figure 5A), xenograft growth was markedly reduced in mice that received C188-9 (50mg/kg/day; Figure 5B, p=0.027). The ability of each compound to inhibit tumor growth correlated with its ability to reduce levels of pSTAT3 within the tumors. Levels of pSTAT3 in tumors from mice treated with C188 were not reduced significantly (Figure 5C, 5D) but pSTAT3 levels were reduced significantly in tumors from mice treated with C-188-9 by 57% (Figure 5E, 5F; p =0.017).

### C188-9 targets both STAT3- and STAT1-regulated genes in UM-SCC-17B xenografts

To determine the effect of C188-9 treatment on STAT3 gene targets, especially pro-oncogenic genes [5, 26], we isolated total RNA from tumor xenografts harvested from mice treated with vehicle (n=5), C188 (n=4), or C188-9 (n=6) and used it for RNA sequencing and analysis (Supplementary Table S3). Of the approximately 13,528 discernible genes, levels of 37 gene transcripts were altered by C188 (17 down and 20 up-regulated, fdr <0.01, fold change ≥ 1.5), of which 7 were known STAT3 gene targets (Supplementary Table S4). In comparison, C188-9 affected a much greater number of genes involved in oncogenesis (384 total, 95 down- and 289 up-regulated), including 76 genes previously reported as regulated by STAT3 (38 down-regulated and 38 up-regulated; Table 3). Among the 38 genes previously shown to be upregulated by STAT3, 24 (63%) genes were downregulated by C188-9 treatment, as expected. Unexpectedly, however, 14 genes (37%) were downregulated by C188-9, including OASL, IFIT3, MX2, and IRF7, previously reported to be negatively regulated by STAT3 (Table 3). Further analysis revealed that many of these 14 genes were reported to be positively regulated by STAT1, as were 16 of the 24 genes previously reported to be upregulated by STAT3. Additionally, we found 10 more genes downregulated by C188-9 (fdr <0.01, fold change ≥ 1.5. Table 3) that previously were shown to be upregulated by STAT1. Thus, 40 of 48 (83.3%) genes downregulated by C188-9 previously were shown to be positively regulated by STAT1 (Table 3), including sixteen genes shown to be co-regulated by STAT3 and STAT1. Many of these genes have been implicated in radioresistance. This analysis raised the possibility that the effect of C188-9 on gene transcript levels in HNSCC tumors was mediated by its effects on both STAT3 and STAT1.

**Table 3:** Known STAT3/1 regulated genes regulated by C188-9 treatment of UM-SCC-17B xenografts in nude mice

Sl	Gene	ID	Description	Fold Change	Regulation by STAT3	Regulation by IFN/STAT1	References
**Downregulated Genes**							
1	SPOCK3	NM_001251967.1	sparc/osteonectin, cwcv and kazal-like domains proteoglycan (testican) 3	−5.1	Pos	-	[52]
2	SFRP1	NM_003012.4	secreted frizzled-related protein 1	−2.6	Pos	-	[53]
3	UPK1B	NM_006952	uroplakin 1B	−1.9	Pos	-	[54]
4	SCARA3	NM_182826.1	scavenger receptor class A, member 3	−1.8	Pos	-	[55]
5	CALML3	NM_005185.2	calmodulin-like 3	−1.7	Pos	-	[53]
6	MMP10	NM_002425	matrix metallopeptidase 10 (stromelysin 2)	−1.7	Pos	-	[56]
7	SLPI	NM_003064	secretory leukocyte peptidase inhibitor	−1.6	Pos	-	[57]
8	CCND3	NM_001081636	similar to Cyclin D3; cyclin D3	−1.6	Pos	-	[58]
9	IFIT1	NM_001548	interferon-induced protein with tetratricopeptide repeats 1	−2.5	Pos	Pos	[20, 59, 60]
10	ISG15	NM_005101	ISG15 ubiquitin-like modifier	−2.3	Pos	Pos	[60, 61]
11	NNMT	NM_006169	nicotinamide N-methyltransferase	−2.3	Pos	Pos	[62, 63]
12	OAS1	NM_002534	2,5-oligoadenylate synthetase 1, 40/46kDa	−2.1	Pos	Pos	[20, 53, 59, 64]
13	IFI6	NM_022873	interferon, alpha-inducible protein 6	−2.1	Pos	Pos	[11, 65]
14	USP18	NM_017414	ubiquitin specific peptidase 18	−1.9	Pos	Pos	[20, 64, 66]
15	MX1	NM_002462	myxovirus (influenza virus) resistance 1, interferon-inducible protein p78 (mouse)	−1.9	Pos	Pos	[20, 53, 62]
16	OAS2	NM_016817	2-5-oligoadenylate synthetase 2, 69/71kDa	−1.8	Pos	Pos	[59, 60]
17	IFI27	NM_005532	interferon, alpha-inducible protein 27	−1.7	Pos	Pos	[53, 62, 67]
18	DDX58	NM_014314	DEAD (Asp-Glu-Ala-Asp) box polypeptide 58	−1.7	Pos	Pos	[60, 68]
19	CLU	NM_001171138	clusterin	−1.7	Pos	Pos	[69, 70]
20	SERPINB3	NM_006919	serpin peptidase inhibitor, clade B (ovalbumin), member 3	−1.6	Pos	Pos	[67, 71]
21	IFITM1	NM_003641	interferon induced transmembrane protein 1	−1.5	Pos	Pos	[53, 72]
22	IFI35	NM_005533	interferon-induced protein 35	−1.9	Pos	Pos	[20, 62, 73]
23	PSMB9	NM_002800	proteasome (prosome, macropain) subunit, beta type, 9 (large multifunctional peptidase 2)	−1.6	Pos	Pos	[62, 73, 74]
24	IFITM3	NM_021034	interferon induced transmembrane protein 3	−1.5	Pos	Pos	[75, 76]
25	OASL	NM_003733	2-5-oligoadenylate synthetase-like	−2.6	Neg	Pos	[61, 67]
26	IFIT3	NM_001549	interferon-induced protein with tetratricopeptide repeats 3	−2.5	Neg	Pos	[59, 67]
27	IFI44L	NM_006820	interferon-induced protein 44-like	−2.3	Neg	Pos	[59, 64, 67]
28	PLSCR1	NM_021105	phospholipid scramblase 1	−1.8	Neg	Pos	[20, 62, 67]
29	MX2	NM_002463	myxovirus (influenza virus) resistance 2 (mouse)	−1.8	Neg	Pos	[77]
30	HERC5	NM_016323	hect domain and RLD 5	−1.7	Neg	Pos	[67, 78]
31	IRF7	NM_004029	interferon regulatory factor 7	−1.7	Neg	Pos	[20, 62, 67]
32	IFI44	NM_006417	interferon-induced protein 44	−1.7	Neg	Pos	[64, 67]
33	TRIM22	NM_006074	tripartite motif containing 22	−1.7	Neg	Pos	[64, 67]
34	SAMD9	NM_017654	sterile alpha motif domain containing 9	−1.7	Neg	Pos	[64, 67]
35	SP110	NM_004509	SP110 nuclear body protein	−1.6	Neg	Pos	[64, 67]
36	HERC6	NM_017912	hect domain and RLD 6	−1.6	Neg	Pos	[67, 79]
37	IFIT5	NM_012420	interferon-induced protein with tetratricopeptide repeats 5	−1.6	Neg	Pos	[67, 80]
38	UBE2L6	NM_004223	ubiquitin-conjugating enzyme E2L 6	−1.5	Neg	Pos	[62, 67]
39	PCDH17	NM_001040429	protocadherin 17	−3.7	-	Pos	[81]
40	CCNA1	NM_001111046	Cyclin A1	−2.3	-	Pos	[20]
41	IFIT2	NM_001547	interferon-induced protein with tetratricopeptide repeats 2	−2.2	-	Pos	[62]
42	EPSTI1	NM_033255	Epithelial Stromal Interaction 1 (Breast)	−2.2	-	Pos	[64]
43	BATF2	NM_138456	basic leucine zipper transcription factor, ATF-like 2	−2.1	-	Pos	[62]
44	CMPK2	NM_207315	cytidine monophosphate (UMP-CMP) kinase 2, mitochondrial	−2.1	-	Pos	[81]
45	GBP1	NM_002053	guanylate binding protein 1, interferon-inducible, 67kDa	−1.7	-	Pos	[62]
46	TYMP	NM_001953	thymidine phosphorylase	−1.7	-	Pos	[82]
47	LY6E	NM_002346	lymphocyte antigen 6 complex, locus E	−1.5	-	Pos	[83]
48	KRT15	NM_002275	keratin 15	−2.1	-	Pos	[84, 85]
**Upregulated Genes**							
1	NPTX2	NM_002523	Neuronal pentraxin II	6.5	Neg	-	[86]
2	SLC2A3	NM_006931	solute carrier family 2 (facilitated glucose transporter), member 3	5.9	Neg	-	[86]
3	CCL2	NM_002982	chemokine (C-C motif) ligand 2	5.1	Neg	-	[86]
4	PTGS2	NM_000963	prostaglandin-endoperoxide synthase 2 (prostaglandin G/H synthase and cyclooxygenase)	4.4	Neg	-	[86]
5	ANPEP	NM_001150	alanyl (membrane) aminopeptidase	3.7	Neg	-	[86]
6	IGFBP3	NM_000598	insulin-like growth factor binding protein 3	3.3	Neg	-	[1]
7	CXCL3	NM_002090	Chemokine (C-X-C motif) ligand 3	2.6	Neg	-	[86]
8	TNC	NM_011607	tenascin C	2.9	Neg	-	[86]
9	AKAP12	NM_005100	A kinase (PRKA) anchor protein 12	2.9	Neg	-	[86]
10	CXCL2	NM_009140	chemokine (C-X-C motif) ligand 2	2.9	Neg	-	[86]
11	SMAD9	NM_019483	MAD homolog 9 (Drosophila)	2.7	Neg	-	[86]
12	THBS1	NM_003246	thrombospondin 1	2.2	Neg	-	[86]
13	CCL20	NM_004591.2	chemokine (C-C motif) ligand 20	2.1	Neg	-	[53]
14	IER3	NM_003897	Immediate early response 3	2.0	Neg	-	[86]
15	FOS	NM_005252	v-fos FBJ murine osteosarcoma viral oncogene homolog	2.0	Neg	-	[86]
16	VEGFA	NM_001171623	vascular endothelial growth factor A	2.0	Neg	-	[86]
17	EGR1	NM_001964	early growth response 1	2.0	Neg	-	[86]
18	NEDD9	NM_001142393	neural precursor cell expressed, developmentally down-regulated 9	1.9	Neg	-	[86]
19	ATF3	NM_001674	activating transcription factor 3	1.9	Neg	-	[86]
20	FOSB	NM_006732	FBJ murine osteosarcoma viral oncogene homolog B	1.9	Neg	-	[86]
21	PHLDA1	NM_007350	Pleckstrin homology-like domain, family A, member 1	1.8	Neg	-	[86]
22	EREG	NM_001432	Epiregulin	1.8	Neg	-	[86]
23	NOTCH4	NM_010929	Notch gene homolog 4 (Drosophila)	1.8	Neg	-	[86]
24	NR4A2	NM_006186	nuclear receptor subfamily 4, group A, member 2	1.8	Neg	-	[86]
25	STC1	NM_003155	stanniocalcin 1	1.8	Neg	-	[86]
26	SLC4A7	NM_001258379	solute carrier family 4, sodium bicarbonate cotransporter, member 7	1.8	Neg	-	[86]
27	ADM	NM_001124	adrenomedullin	1.8	Neg	-	[86]
28	COL5A1	NM_000093	collagen, type V, alpha 1	1.7	Neg	-	[86]
29	SLC2A1	NM_006516	solute carrier family 2 (facilitated glucose transporter), member 1	1.7	Neg	-	[86]
30	VLDLR	NM_003383	very low density lipoprotein receptor	1.7	Neg	-	[86]
31	PDK1	NM_002610	pyruvate dehydrogenase kinase, isozyme 1	1.7	Neg	-	[86]
32	SERTAD2	NM_014755	SERTA domain containing 2	1.6	Neg	-	[86]
33	HK2	NM_000189	hexokinase 2	1.6	Neg	-	[86]
34	NAV1	NM_173437	neuron navigator 1	1.6	Neg	-	[86]
35	SLC7A11	NM_014331	Solute carrier family 7, (cationic amino acid transporter, y+ system) member 11	1.6	Neg	-	[86]
36	HSPG2	NM_005529.5	heparan sulfate proteoglycan 2	1.5	Neg	-	[26]
37	TGFBR3	NM_011578	transforming growth factor, beta receptor III	1.5	Neg	Neg	[20, 86]
38	ALDH3A1	NM_000691	aldehyde dehydrogenase family 3, subfamily A1	2.6	Pos	-	[86]
39	NRP2	NM_201266	neuropilin 2	1.9	-	Pos	[62]
40	COL16A1	NM_001856	collagen, type XVI, alpha 1	1.8	-	Pos	[62]
41	CYP1B1	NM_000104	cytochrome P450, family 1, subfamily B, polypeptide 1	2.0	-	Pos	[62]
42	DYNC1H1	NM_001376	dynein, cytoplasmic 1, heavy chain 1	1.5	-	Pos	[62]

RNA-sequence data was analyzed as stated in methods. Identification of all differentially expressed genes was based on a cutoff false detection rate fdr < 0.01, and a fold change FC > 1.5. The absolute value of FC is the magnitude of up- or down-regulation for each gene/homolog after C188-9 treatment. FC > 1.5 indicates up-regulation, and < −1.5 indicates down-regulation. The genes in this table are arranged in decreasing order of FC, which are shown in column 5. It also shows literature info on whether a gene is reported to be positively (Pos) or negatively (Neg) regulated by STAT3 (Column 5) and/or STAT1 (Column 6).

To explore the hypothesis that the activity of C188-9 extended to STAT1, we examined the ability of C188-9 to inhibit STAT1. Examination of Kasumi-1 cells revealed that C188-9 is a potent inhibitor of STAT1 activation by IFN-γ (IC_50_=9.5 μM; Table 1, Figure 2B, 2C), as well as by G-CSF (IC_50_ =4.1 μM, Table 1, Figure 2D). In addition, C188-9 was effective at reducing levels of constitutively activated STAT1 in UM-SCC-17B cells (IC_50_=19.1 μM, Table 2) as well as SCC61 and HN30 (IC_50_=28.5 and 5 μM respectively, Table 2) but not in SCC-35, which did not have significantly higher pSTAT1 levels compared to the non-tumor line HEEpiC (Table 2, Supplementary Figure S1B. Importantly, levels of pSTAT1 in UM-SCC-17B tumor xenografts from mice treated with C188-9 were reduced by 80% compared to tumor xenografts from mice that received vehicle control (p = 0.000003; Figure 5E, 5F and Table 1).

## DISCUSSION

We used the scaffold of C188, a small-molecule STAT3 inhibitor previously identified by us using virtual ligand screening, in a hit-to-lead program to identify a more potent small-molecule STAT3 inhibitor. These studies identified C188-9, which binds to STAT3 with high affinity and is more potent than C188 in inhibiting STAT3 binding to its pY-peptide ligand, in inhibiting cytokine-stimulated pSTAT3, in reducing constitutive pSTAT3 activity in UM-SCC-17B, a radioresistant HNSCC cell line, and in inhibiting anchorage dependent and independent growth of these cells. In addition, treatment of nude mice bearing UM-SCC-17B xenografts with C188-9, but not C188, prevented tumor xenograft growth. RNA-seq analysis of tumor xenografts revealed that C188-9 modulated many STAT3-regulated genes involved in oncogenesis, as well as genes involved in chemoresistance and radioresistance that previously were shown to be regulated by STAT3 and STAT1. Phosphoflow and Luminex assays of cells treated with IFN-γ or G-CSF and immunoblotting of lysates of UM-SCC-17B xenografts revealed that C188-9 was equally potent at targeting STAT1 as STAT3. Thus, C188-9, either alone or in combination with radiotherapy, has potential for use in treating HNSCC tumors that demonstrate increased STAT3 or STAT1 activation.

Other small molecule programs directed at identifying drug-like compounds targeting STAT3 homodimer or STAT3 SH2 domain also identified promising hits. The IC_50_ values for STAT3 inhibition by these inhibitors, including Stattic (5.1 μM for inhibition of STAT3 binding to pY-peptide [27]), STA-21 (12.2-18.7 μM for inhibition of STAT3 in a luciferase reporter assay [33]), S3I-201 (86 μM for inhibition of STAT3 DNA binding [28]) and XZH-5 (20-30 μM for inhibition of cytokine-induced pSTAT3 levels [34]) were higher than that of C188-9 (3.7 μM in inhibiting G-CSF-induced pSTAT3 levels, Table 1). The second-generation derivatives of many of these original hits had activity equivalent to or up to 3-fold greater than their parent compound in cancer cell growth-inhibition assays, but the increase in STAT3-inhibitory potency did not always correlate to the anti-proliferative capacity. For example, LLL12, a second-generation STA-21 derivative has an IC_50_ of 0.6 – 3.1 μM (inhibition of cytokine-induced pSTAT3 [35]), a marked improvement, but killed tumor cells only marginally better (IC_50_ ~5 μM [35, 36]). Evidence of a direct effect of LLL12 on STAT3 vs. an upstream kinase is not provided, which may explain this discordance [35, 37]. Second and third generation derivatives of the DNA-binding inhibitor, S3I-201 (IC_50_ of 86 μM [28]), such as S3I-201.1066 (IC_50_=35 μM [38]), and BP-1-102 (IC_50_=6.8 μM [39]) show a stepwise improvement of anti-STAT3 activity. This improvement in inhibition of STAT3 DNA binding was accompanied by increased binding affinity measured by surface plasmon resonance (SPR), which was 2.7 μM for S3I-201.1066 and 504 nM for BP-1-102. But BP-1-102 was also found to affect NF-κB activity, perhaps by affecting cross talk between STAT3 and NF-κB [39].

Several of small-molecule STAT3 inhibitors, as well as other classes of agents, have been shown to be effective in targeting STAT3 and inhibiting tumor growth in preclinical models, including HNSCC, [33, 40]. In particular, Stattic [19], STAT3 anti-sense plasmid [14], STAT3 decoy oligonucleotide [41], erlotinib [42], and most recently, a cyclic version of oligonucleotide decoy [43], have shown promise in pre-clinical models of HNSCC. However, an agent that directly targets STAT3 has yet to be approved by the FDA and it remains to be seen whether C188-9 or any of these agents will progress beyond Phase 0 clinical trials for HNSCC. Towards this end, we demonstrated that C188-9 has a favorable pharmacokinetic and safety profile in mice (Table 1); C188-9 was well tolerated in mice to 100 mg/kg/day for 14 days, demonstrated plasma bioavailability by the oral route similar to the IP route, and was concentrated in tumors to levels nearly 3-fold that of simultaneously harvested plasma. Also, in GLP-compliant safety studies, C188-9 demonstrated no clinical, laboratory, or pathological abnormalities in rats or dogs up to a dose of 200 mg/kg/d or 100 mg/kg/d, respectively, for 28 days (data not shown).

In addition to promoting many hallmarks and enablers of cancer, STAT3 activation has been linked to resistance to radiation therapy in cells obtained from several normal tissues and cancers, including HNSCC [19], through several mechanisms, including EGFR signaling [44], HER2-STAT3 cross-talk [45], and activation of the LIF-STAT3 axis [46]. Addition of STAT3 inhibitors [19, 47] to radiation therapy increased clearance of cancer xenografts in mice. Thus, we were not surprised to find modulation of STAT3-regulated genes shown previously to contribute to resistance to radiation within tumor xenografts of mice treated with C188-9 vs. vehicle. However, RNAseq analysis of these tumor xenografts also uncovered modulation of many STAT1-regulated genes, including many, involved in radiation resistance, raising the possibility that C188-9 targeted STAT1, in addition to STAT3, in these tumors. In fact, examination of the effects of C188-9 on STAT1 in each of our *in vitro* assays, as well as in tumor xenografts, demonstrated that C188-9 was as effective at targeting STAT1 as it was in targeting STAT3. This is not entirely surprising, given the high degree of similarity between the SH2 domains of STAT1 and STAT3 [29].

The role of increased STAT1 activity in tumors resistant to IR [20, 48], as well as chemotherapy e.g. doxorubicin and topoisomerase-II inhibitors [48], now is well established. Khodarev *et al* developed an IR-resistant, HNSCC cell line, Nu-61, from a xenograft in nude mice that grew out after multiple rounds of implantation and irradiation of xenografts starting with the IR-sensitive HNSCC cell line, SCC-61 [20]. Comparison of gene expression profiles of the two lines, established that the IFN/STAT1 pathway is responsible for this acquired IR resistance. A signature list of 25 such genes, termed the IFN-related damage signature (IRDS [23]), was also shown to be induced by IR therapy in xenograft tumor models of head and neck, breast, and colon cancer [21]. Furthermore, STAT1 silencing in IR-resistant Nu-61 cells rendered them IR sensitive with concurrent reduction of these IRDS genes [21, 23]. Our RNA-seq data shows that the same set of signature genes is upregulated in xenografts of UM-SCC-17B, a cell line derived form a tumor from a patient who failed multiple rounds of radiotherapy [49]. These findings suggest that adjuvant use of C188-9, a dual inhibitor of STAT1 and STAT3 in HNSCC, may overcome IR resistance in this tumor system. Recently, both STAT3 and STAT1 were discovered among the top transcription factors activated in IR-resistant HPV-negative HNSCC and combined siRNA mediated inhibition of STAT3 and STAT1 had more pronounced effect on cell growth of STAT-activated H0N1 cells [50]. Our study describing the dual anti-STAT3 and anti-STAT1 action of C188-9 on HNSCC cells with activated STAT3/1 thus becomes particularly relevant.

## MATERIALS AND METHODS

### Cell lines

HNSCC cell lines SCC-9, SCC-15, HN5, UM-SCC-1, HN30 and HN31 (obtained from Dr. Heath Skinner, at MDA), SCC-61, SQ-20B, SCC-35 (obtained from Dr. Ralph Weichselbaum, Department of Radiation Oncology, University of Chicago, Chicago, IL) and UM-SCC-17B (obtained from Dr. Thomas E Carey from University of Michigan) were genotyped at 14 loci for authentication by the cell-typing core at MDACC. SCC-9, SCC-15, HN5, UM-SCC-1 were maintained in 5% CO_2_ chambers at 37°C in DMEMF12 with 10% FBS and UM-SCC17B, HN30 and HN31 in DMEM with 10% FBS plus other common additives. SCC-61, SQ-20B and SCC-35 were maintained in 7% CO2 chambers in DMEMF12 with 20% FBS and other additives. The primary esophageal line, Human Esophageal Epithelial Cells (HEEpiC), were obtained (http://www.sciencellonline.com/OLDSITE/site/productInformation.php?keyword=2720) from the company ScienCell and maintained in its specific medium the Epithelial Cell Medium-2 (EpiCM-2) and was used within one month of first culturing it.

### 2-D fingerprint screening

Using C188 scaffold as a reference structure, 2-D fingerprint screening was performed using the Life Chemicals database, which contained over 490,000 compounds. We used 100 percent similarity as cutoff and employed Tanimoto coefficient/Unity/Sybyl/Tripos as the similarity comparing method.

### 3-D pharmacophore sorting

The 207 compounds resulted from 2-D fingerprint screening were converted into 3-D structures for analysis as described in Supplemental Information.

### QSAR studies

3-D quantitative structure activity relationship (QSAR) study was performed as described in Supplemental Information.

### STAT3/pY-peptide binding using a surface plasmon resonance (SPR) assay

Binding of STAT3 (200nM in 20 mM Tris buffer, pH 8) pre-incubated without or with C188/C188-9 to phosphorylated and control non-phosphorylated biotinylated EGFR derived dodecapeptides based on sequence surrounding Y1068 [51] was measured using a Biacore 3000 biosensor (Biacore inc., Piscataway NJ) as described in Supplemental Information.

### Phosphoflow assay

Phosphoflow analysis was done as described in Xu et al [29]. Briefly, Kasumi-1 cells, were serum-starved, pre-treated with compound or DMSO (1 hour, RT) and then stimulated with 20 μl of GCSF (10 ng/ml) for 15 minutes at 37°C. The cells were then permeabilized and stained with Alexa647-pSTAT3, Alexa488-pSTAT5 and PE-pSTAT1 antibodies and analyzed using the BD LSR2. FCS files were exported and uploaded to Cytobank for determination of phosphoproteins as earlier [5]. Gating was done on live, single cells and then gates selected for positive ligand-activated pSTAT3 and pSTAT1. IC_50_ values were calculated using the GraphPad Prism.

### Luminex bead-based assay

Luminex bead-based assays were used to determine levels of pSTAT1, pSTAT3, and GAPDH, as described [5] as detailed in Supplemental Information.

### Microscale thermophoresis

Binding of C188-9 or EGFR Tyr(P)-1068 phosphopeptide to WT STAT3β (127-722) was measured by microscale thermophoresis (MST). C188-9 was titrated between 0.305 and 10,000 nM and EGFR Tyr(P)-1068 was titrated between 0.025 and 800 nM to a constant amount (~80 nM) of fluorescently labeled STAT3 (127-722). Movement of STAT3 under a temperature gradient was measured by recording the change of fluorescence signal as the heated molecules moved away from the point of application of the IR-laser used for heating. This movement was traced in the fluorescence time trace. The change in fluorescence F_norm_=F_hot_/F_cold_ where F_cold_ is the homogeneous fluorescence distribution observed inside the capillary before the IR-Laser is switched on and F_hot_ is the steady low fluorescence state after the IR-Laser is switched on for 30s. F_norm_ was calculated and plotted against the logarithm of the different concentrations of the peptide or C188-9 dilution series, to obtain a sigmoidal binding curve. This binding curve was fitted with the nonlinear solution of the law of mass action, and the dissociation constant K_D_ was calculated.

### Anchorage-independent and dependent cell growth

Cells were cultured in triplicates in in complete DMEM ± drug, in ultra-low attachment 96 well plates for 72 hrs or cell-culture treated plates for 48 hrs and viable cells were quantitated using MTT. Optical density (OD) was measured at 590 nm using a 96-well multi-scanner (EL-800 universal microplate reader, BioTek Inc, VT, USA). Relative % viability (viability after any treatment ÷ viability of untreated cells × 100) was plotted along Y-axis. At least 2 replicates experiments were performed and were used for IC50 calculation using GraphPad software.

### UM-SCC-17B xenografts

UM-SCC-17B cells (1.5 × 10^6^) were injected into the tongues of athymic, 8–10 week old, male, nude mice (NCI, Frederick, MD, USA). Once tumors were established, mice (20 total; 10/group) were randomized (average tumor vol ~ 15 – 20 mm^3^) to receive 5 times a week, intraperitoneal injections of either DMSO or C188 (50 mg/Kg) or C188-9 (100 mg/Kg). Tumor volumes were measured twice weekly. Average tumor volumes 6/π × (long dimension) × (short dimension)^2^) were calculated and normalized to the volume at first day of treatment and plotted Comparison was done by t test (* p<0.05).

### RNA extraction and sequencing using RNA-seq

Tissue was cryosliced (5μ) with Cryotome and total RNA was extracted using RNeasy Microarray tissue kit (Qiagen, Venlo, Limburg) followed by DNase digestion and Qiagen RNeasy column purification (Qiagen, Valencia, CA, USA). The RNA integrity was verified using an Agilent Bioanalyzer 2100 (Agilent, Palo Alto, CA, USA). High-quality RNA (RNA Integrity number or RIN >9.0) was processed using an Illumina TruSeq RNA sample prep kit following the manufacturer's instruction (Illumina, San Diego, CA, USA). Detailed method is provided in Supplemental Info.

### PK studies in mice

Pharmacokinetic (PK) studies to assess plasma levels of C188-9 following IP and oral routes were performed in C57BL/6 mice. PK studies to assess tumor vs. plasma levels of C188-9 were performed in nude mice bearing xenograft tumors. Details regarding these studies are in the Supplemental Methods.

### Statistical analysis

Student's t-test or a paired t-test was used to compare control- and C188-9 treated groups as indicated.

## SUPPLEMENTARY INFORMATION FIGURE AND TABLES




