# Correction: Small-molecule inhibition of STAT3 in radioresistant head and neck squamous cell carcinoma

**DOI:** 10.18632/oncotarget.26743

**Published:** 2019-02-22

**Authors:** Uddalak Bharadwaj, T. Kris Eckols, Xuejun Xu, Moses M. Kasembeli, Yunyun Chen, Makoto Adachi, Yongcheng Song, Qianxing Mo, Stephen Y. Lai, David J. Tweardy

**Affiliations:** ^1^ Department of Infectious Disease, Infection Control and Employee Health, The University of Texas MD Anderson Cancer Center, Houston, Texas, USA; ^2^ The Key Laboratory of Natural Medicine and Immuno-Engineering, Henan University, Kaifeng, China; ^3^ Department of Head and Neck Surgery, Division of Surgery, The University of Texas MD Anderson Cancer Center, Houston, Texas, USA; ^4^ Department of Pharmacology, Baylor College of Medicine, Houston, Texas, USA; ^5^ Department of Medicine, Division of Biostatistics, Dan L. Duncan Cancer Center, Section of Hematology/Oncology, Baylor College of Medicine, Houston, Texas, USA; ^6^ Department of Molecular & Cellular Oncology, The University of Texas MD Anderson Cancer Center, Houston, Texas, USA

**This article has been corrected:** The correct dose for the compund of C188-9 in results section is given below:

We next examined the effect of C188 and C188-9 on growth of tumor xenografts, which revealed that the greater growth inhibitory activity of C188-9 vs. C188 extended to UM-SCC-17B cell line xenografts. While established UM-SCC-17B xenograft tumors continued to grow in nude mice that received C188 (50 mg/kg/day; Figure 5A), xenograft growth was markedly reduced in mice that received C188-9 (100 mg/kg/day; Figure 5B, p=0.027). The ability of each compound to inhibit tumor growth correlated with its ability to reduce levels of pSTAT3 within the tumors. Levels of pSTAT3 in tumors from mice treated with C188 were not reduced significantly (Figure 5C, 5D) but pSTAT3 levels were reduced significantly in tumors from mice treated with C-188-9 by 57% (Figure 5E, 5F; p=0.017).

Original article: Oncotarget. 2016; 7:26307-26330. https://doi.org/10.18632/oncotarget.8368

