# Supplementary material for: Small-molecule inhibition of STAT3 in radioresistant head and neck squamous cell carcinoma
Source: Oncotarget. 2016 Mar 25;7(18):26307–30. doi: 10.18632/oncotarget.8368 (PMC5041982; doi:10.18632/oncotarget.8368)
Supplement: Supplementary file 1 [file oncotarget-07-26307-s001.pdf]

SUPPLEMENTARY INFORMATION

3-D pharmacophore sorting

The 207 compounds that resulted from 2-D fingerprint screening were converted into 3-D structures for analysis as described below.

3-D pharmacophore generation

Each molecular chemical feature, including hydrogen bond donor, hydrogen bond acceptor, positive and negative ionizable group, and hydrophobic region, was generated using the LigandScout application framework and projected into 3-D space [15].

Feature-pairing

Each chemical feature was converted into distance bin and the complete set of distance bins used to construct a cost matrix. The most favorable pairing based on cost matrix was identified.

3-D pharmacophore transformation

A Kabsch matrix was applied to determine the optimal rotation and translation. The best alignment was obtained after multiple cycles of feature pairing with transformation based on orthogonal matrix normalization. We ranked each of the 207 compounds in decreasing order of pharmacophore similarity to C188 and purchased the top 39 compounds, each with a pharmacophore score over 70, for laboratory testing.

STAT3/pY-peptide binding assay

STAT3 binding assays were performed at 25ºC with a BIAcore 3000 biosensor using 20 mM Tris buffer pH 8 containing 2 mM mercaptoethanol and 5% DMSO as the running buffer [9]. Phosphorylated and control non-phosphorylated biotinylated EGFR derived dodecapeptides based on the sequence surrounding Y1068 [11] were immobilized on a streptavidin coated sensor chip (BIAcore inc., Piscataway NJ). The binding of STAT3 was conducted in 20 mM Tris buffer pH 8 containing 2 mM β-mercaptoethanol at a flow rate of 10 µL/min for 1-2 minute. Aliquots of STAT3 at 500 nM were premixed with compound to achieve a final concentration of 1-1,000 µM and incubated at 4°C prior to being injected onto the sensor chip. The chip was regenerated by injecting 10 µL of 100 mM glycine at pH 1.5 after each sample injection. A control (STAT3 with DMSO but without compound) was run at the beginning and the end of each cycle (40 sample injections) to ensure that the integrity of the sensor chip was maintained throughout the cycle run. The average of the two controls was normalized to 100% and used to evaluate the effect of each compound on STAT3 binding. Responses were normalized by dividing the value at 2 min by the response obtained in the absence of compounds at 2 min and multiplying by 100. IC_{50} values were determined by plotting % maximum response as a function of log concentration of compound and fitting the experimental points to a competitive binding model using a four parameter logistic equation: $R = R_{high} - (R_{high} - R_{low})/ (1 + conc/A1)^A2$, where R = percent response at inhibitor concentration, R_{high} = percent response with no compound, R_{low}= percent response at highest compound concentration, A2 = fitting parameter (slope) and A1 = IC_{50} (BIAevaluation Software version 4.1). The inhibition constant (Ki) was derived from the Cheng-Prusoff equation $Ki=IC_{50}/(1+[C]/K_d)$ as used by Halling et al [7] where C stands for STAT3 concentration used in the SPR assay and K_d denotes the dissociation constant for STAT3 binding to its phosphorylated EGFR ligand in the SPR assay (9.5 nM).

Luminex bead-based assay

A Luminex bead-based assay was used to determine levels of pSTAT1 and pSTAT3, and GAPDH, as described [5]. Briefly, Kasumi-1 cells, were serum-starved, pre-treated with compound (0.1/0.3/1/3/10/30/100 mM) or DMSO and then stimulated with 20 µl of G-CSF (10 ng/ml) for 15 minutes at 37°C. The assay was stopped by ice-cold PBS, and total protein extracted from cell pellets. Total protein was plated in a 96-well filter plate pre-loaded with beads (Millipore, Danvers, MA) coupled to antibody against pSTAT antibodies and GAPDH. Bead-bound analytes were measured using biotinylated detection antibody specific for a different epitope and streptavidin-phycoerythrin (streptavidin-PE) [4, 8]. Constitutive pSTAT1/3 activities were also measured in lysates made from non-synchronous cultures of HNSCC cell line UM-SCC-17B, in a similar manner. Data were collected and analyzed using the Bio-Plex suspension array system (Luminex 100 system, Bio-Rad Laboratories, Hercules, CA). GAPDH-normalized pSTAT3 values from each treatment were corrected for untreated cells, expressed as percentage untreated, and used to determine the IC_{50} using GraphPad.

Microscale thermophoresis

Binding of C188-9 and EGFR Tyr(P)-1068 phosphopeptide to WT Stat3β (127-722) was measured by Microscale thermophoresis (MST). Stat3 was labeled using the Monolith NT protein Labeling kit

(NanoTemper Technologies, Munchen, Germany). C188-9 was titrated between 0.305 and 10,000 nM and EGFR Tyr(P)-1068 was titrated between 0.025 and 800 nM to a constant amount (~80 nM) of fluorescently labeled Stat3 (127-722). Movement of Stat3 under a temperature gradient was measured by recording the loss of fluorescence as the heated molecules moved away from the point of application of the IR-laser used for heating. This movement was traced in the fluorescence time trace. The change in fluorescence $F_{norm}=F_{hot}/F_{cold}$ where F_{cold} is the homogeneous fluorescence distribution observed inside the capillary before the IR-Laser is switched on and F_{hot} is the steady low fluorescence state after the IR-Laser is switched on for 30s. F_{norm} was calculated and plotted against the logarithm of the different concentrations of the peptide/C188-9 dilution series, to obtain a sigmoidal binding curve. This binding curve was fitted with the nonlinear solution of the law of mass action, and the dissociation constant K_D was calculated.

QSAR studies

3-D quantitative structure activity relationship (QSAR) study was performed as described [6], using the Phase [1] program in Schrödinger (version 2010) [2]. Compounds were built, energy and geometry minimized using the OPLS-2005 force field in Maestro [3] in Schrödinger. They were then aligned using the “flexible ligand alignment” module in Maestro. The aligned ligands were imported into the Phase program. Partial least squares (PLS) method was applied to correlate the STAT3 inhibitory activities (pIC50) of these compounds with the Phase field data calculated based on their 3-D structures to generate a QSAR model. Five leave-5-out training/test sets were performed to validate the QSAR model. 3-D QSAR visualization was generated using Phase.

Immunoblot assay

Tumor tissue was minced, homogenized and proteins extracted using high-salt buffer, as described [12]. Proteins (20 mg) were separated by 7.5% SDS-PAGE and transferred to polyvinylidene fluoride (PVDF) membrane (Millipore, Waltham, MA) and immunoblotted. Membranes were probed with antibody against STAT1 pY^{701} or STAT3 pY^{705} total STAT1 or STAT3 (Transduction labs, Lexington, KY) and β–actin (Abcam, Cambridge, MA). Membranes were stripped between antibody probing using Restore™ Western Blot Stripping Buffer (Thermo Fisher Scientific Inc., Waltham, MA). Horseradish peroxidase-conjugated goat-anti-mouse IgG was used as the secondary antibody (Invitrogen Carlsbad, CA) and the membranes were developed with enhanced chemiluminescence (ECL) detection system (Amersham Life Sciences Inc.; Arlington Heights, IL.).

RNA extraction and sequencing using RNA-seq

Tissue was cryosliced (5m) with Cryotome and total RNA was extracted using RNeasy Microarray tissue kit (Qiagen, Venlo, Limburg) followed by DNase digestion and Qiagen RNeasy column purification (Qiagen, Valencia, CA, USA). The RNA integrity was verified using an Agilent Bioanalyzer 2100 (Agilent, Palo Alto, CA, USA). High-quality RNA (RNA Integrity number or RIN >9.0) was processed using an Illumina TruSeq RNA sample prep kit following the manufacturer’s instruction (Illumina, San Diego, CA, USA). Detailed method is provided in Supplemental Info. After quality control procedures, individual RNA-seq libraries were then pooled based on their respective 6-bp adaptors and sequenced at 50 bp/sequence, read using an Illumina HiSeq 2000 sequencer, as described previously [10]. Approximately 67.5 million reads per sample (mean ± sd = 67,527,111 ± 8,330,388.6) were generated.

RNA-Seq data analysis and bioinformatics

The quality of RNA-seq reads was first checked using FastQC software. Low quality nucleotides from 5’ and 3’ ends of reads were trimmed and then aligned to the human reference genome (UCSC hg19) using TopHat [13] with NCBI RefSeq genes as the reference. The Cuffdiff program in the Cufflink package (v2.1.1) [14] was used to test for differential expression between groups of interest with the default parameters.

PK studies in mice

Sample collection

For the pharmacokinetic studies, C188-9 was formulated as for IP and PO dosing in 10% Ethanol (added first), 50% PEG-400 (added second), 40% Saline (added last) then vortexed and sonicated at concentration (10 mg/Kg, 1 mg/mL, 10 mL/kg). The dosing solution was freshly made on the dosing day. C57BL/6 mice were provided in triplicate, a single dose by body weight via either intra-peritoneal (IP) or oral (PO) route at 10 mg/kg at a volume of 10 mL/kg and plasma samples collected as shown in the study table below. At the indicated times, 3 mice per time point were euthanized per dose route and then blood collected via cardiac puncture. Blood was collected in K2EDTA blood tubes then inverted and centrifuged at 6000rpm for 5min in 4°C before being decanted. All plasma samples were stored at -80°C until analysis. Plasma for calibration curve was also prepared similarly.

Timetable for plasma collection:

Agent	Route	Type of mouse	# of Mice	Dose (mg/Kg)	Sample Collected	Collection time points
188-9	IP	CD-1	24	10	Plasma	0, 0.25, 0.5, 1, 2, 4, 8 and 24 hours
188-9	PO	CD-1	24	10	Plasma	0, 0.25, 0.5, 1, 2, 4, 8 and 24 hours

For the tumor accumulation study, $3x10^6$, MDA-MB 468 cells were sub-cutaneously injected on left flank of Nude (Athymic NCr-nu/nu) mice and allowed to grow till 500 mm^3 and then the mice were randomized into two groups, Control and C188-9 (10mg/Kg) treatment group. The Control mice (n =2) were sacrificed after their tumor reached 500 mm^3 and the mice in the treatment group (n = 5) were treated with C188-9 at 10 mg/Kg, IP, twice daily (8AM, 8 PM) for 4 days. On day 5, a final dose was administered and mice were sacrificed an hour later. Plasma and tumors were harvested as described before, snap frozen and shipped to Apredica for analysis.

Sample analysis

Plasma samples were processed with 3 volumes of methanol containing internal standard. Samples were then centrifuged to remove precipitated protein, and the supernatant was analyzed by LC/MS-MS. All plasma samples were compared to a calibration curve prepared in mouse blank plasma. Tumor samples were first homogenized in 1 ml of PBS per gram of tumor tissue and then processed in the same manner as the plasma samples. Tumor samples were quantified using a calibration curve prepared in control tumor.

Samples were analyzed by LC/MS/MS using an Agilent 6410 mass spectrometer coupled with an Agilent 1200 HPLC and a CTC PAL chilled autosampler, all controlled by MassHunter software (Agilent). After separation on a C18 reverse phase HPLC column (e.g. Zorbax Eclipse 2.1mmx50mm or Zorbax Poroshell 2.1x50mm, Agilent, Waters, or equivalent) using an acetonitrile-water gradient system, peaks were analyzed by mass spectrometry (MS) using ESI ionization in MRM mode.

REFERENCES

1. *Phase*, 3.2 edn: New York, 2010.
2. Scrodinger Suite, version 2010, Schrodinger, LLC, New York, NY, 2010, 2010.
3. Maestro, version 9.1, LLC, New York, NY, 2010, 2010.
4. Aktas B, Tewes M, Fehm T, Hauch S, Kimmig R, Kasimir-Bauer S. Stem cell and epithelial-mesenchymal transition markers are frequently overexpressed in circulating tumor cells of metastatic breast cancer patients. Breast Cancer Res 2009; 11: R46.
5. Bharadwaj U, Eckols TK, Kolosov M, Kasembeli MM, Adam A, Torres D *et al*. Drug-repositioning screening identified piperlongumine as a direct STAT3 inhibitor with potent activity against breast cancer. Oncogene 2014; 0.
6. Deng L, Diao J, Chen P, Pujari V, Yao Y, Cheng G *et al*. Inhibition of 1-deoxy-D-xylulose-5-phosphate reductoisomerase by lipophilic phosphonates: SAR, QSAR, and crystallographic studies. J Med Chem 2011; 54: 4721-4734.
7. Halling DB, Georgiou DK, Black DJ, Yang G, Fallon JL, Quiocho FA *et al*. Determinants in CaV1 channels that regulate the Ca2+ sensitivity of bound calmodulin. J Biol Chem 2009; 284: 20041-20051.
8. Kim BK, Lee JW, Park PJ, Shin YS, Lee WY, Lee KA *et al*. The multiplex bead array approach to identifying serum biomarkers associated with breast cancer. Breast Cancer Res 2009; 11: R22.
9. Kim JK, Xu Y, Xu X, Keene DR, Gurusiddappa S, Liang X *et al*. A Novel Binding Site in Collagen Type III for Integrins {alpha}1{beta}1 and {alpha}2{beta}1. J Biol Chem 2005; 280: 32512-32520.
10. Li RW, Schroeder SG. Cytoskeleton remodeling and alterations in smooth muscle contractility in the bovine jejunum during nematode infection. Funct Integr Genomics 2012; 12: 35-44.
11. Shao H, Xu X, Mastrangelo M-AA, Jing N, Cook RG, Legge GB *et al*. Structural Requirements for Signal Transducer and Activator of Transcription 3 Binding to Phosphotyrosine Ligands Containing the YXXQ Motif. J Biol Chem 2004; 279: 18967-18973.
12. Shao H, Xu X, Jing N, Tweardy DJ. Unique Structural Determinants for Stat3 Recruitment and Activation by the Granulocyte Colony-Stimulating Factor Receptor at Phosphotyrosine Ligands 704 and 744. J Immunol 2006; 176: 2933-2941.
13. Trapnell C, Pachter L, Salzberg SL. TopHat: discovering splice junctions with RNA-Seq. Bioinformatics 2009; 25: 1105-1111.
14. Trapnell C, Williams BA, Pertea G, Mortazavi A, Kwan G, van Baren MJ *et al*. Transcript assembly and quantification by RNA-Seq reveals unannotated transcripts and isoform switching during cell differentiation. Nat Biotechnol 2010; 28: 511-515.
15. Wolber G, Langer T. LigandScout: 3-D Pharmacophores Derived from Protein-Bound Ligands and Their Use as Virtual Screening Filters. J Chem Inf Model 2005; 45: 160-169.

SUPPLEMENTARY FIGURE AND TABLES

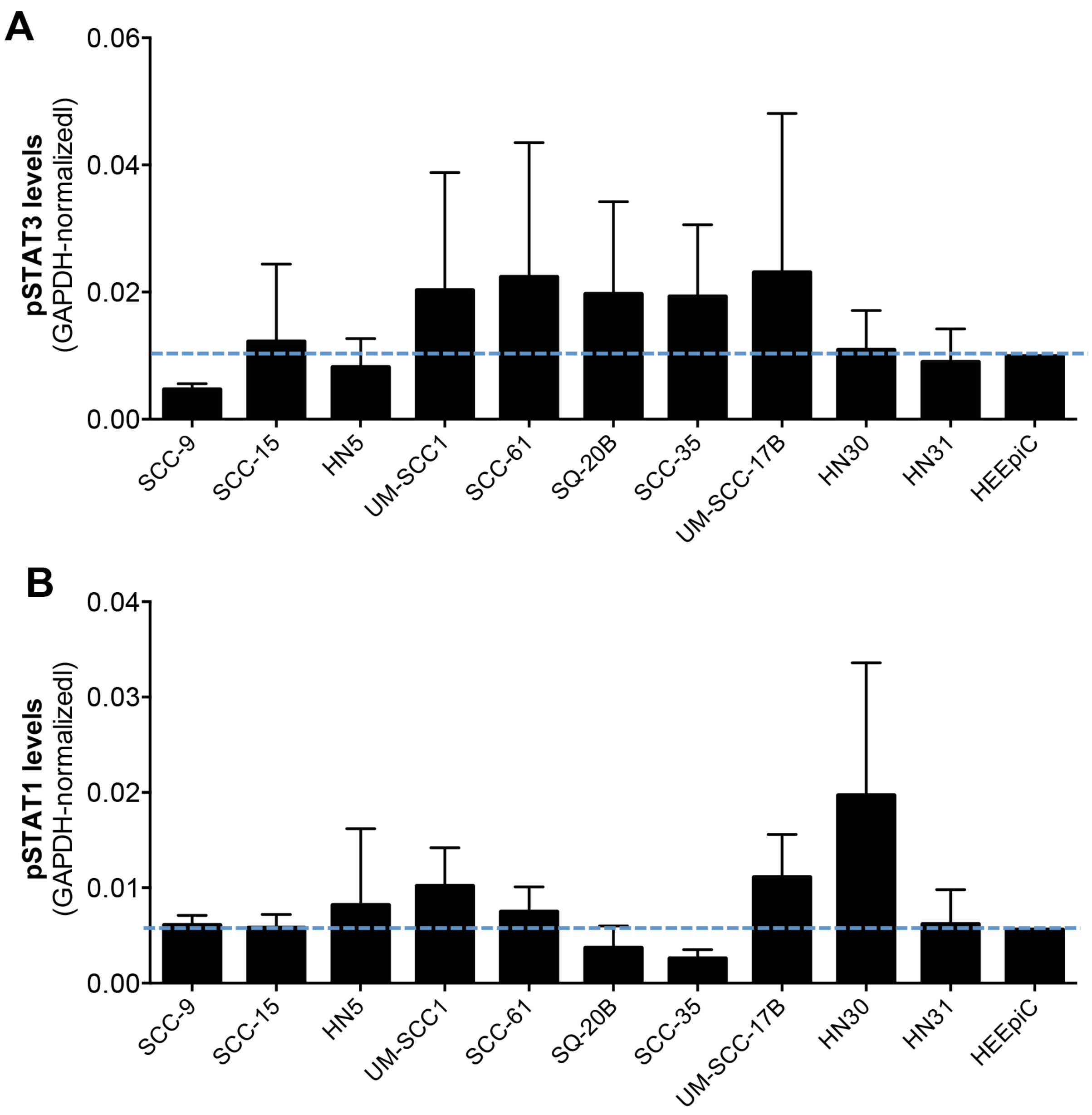

Supplementary Figure S1: Luminex beads were used to test lysates from asynchronous cultures of HNSCC cell lines SCC-9, SCC-15, HN5, UM-SCC-1, SCC-61, SQ-20B, SCC-35, UM-SCC-17B, HN30 and HN31 (4 replicate experiments) and the primary Human Esophageal Epithelial Cells (HEEpiC) for pSTAT3/pSTAT1 and GAPDH. GAPDH-normalized pSTAT3 **A.** or pSTAT1 **B.** values are plotted. Blue dotted line denotes the expression level in the non-tumor line HEEpiC.

Supplementary Table S1: Summary of STAT inhibitory activities (IC_{50} ± SD, n ≥ 2) of C188 and 39 other compounds obtained by 2D similarity screening and 3D pharmacophore search of chemical libraries.

See Supplementary File 1

Supplementary Table S2: QSAR statistics for STAT3 inhibition using Phase

	training set	test set 1	test set 2	test set 3	test set 4	test set 5
r^2	0.94	0.93	0.95	0.92	0.90	0.94
q^2	0.58	0.59	0.59	0.57	0.59	0.60
Standard deviation	0.19	0.21	0.17	0.23	0.24	0.20
F-test	133.0	142.1	202.1	122.2	95.8	127.6
N	4	3	3	3	3	4
n	40	35	35	35	35	35

Supplementary Table S3: Genes regulated by C188 and C188-9 treatment of UM-SCC-17B xenografts in nude mice

	**C188/Control** fdr< 0.01, FC ≥ 1.5			**C188-9/Control** fdr< 0.01, FC ≥ 1.5		
	**Down regulated**	**Up regulated**	**Total**	**Down regulated**	**Up regulated**	**Total**
**STAT1/3 regulated**	7	1	**8**	48	42	**90**
**Others**	10	19	**29**	47	247	**294**
**Total**	**17**	**20**	**37**	**95**	**289**	**384**

Supplementary Table S4: Known STAT3/1 regulated genes affected by C188 treatment of UM-SCC-17B xenografts in nude mice

Gene	GeneID	Descripton	Fold Regulation	Regulation by STAT3	Regulation by IFN/STAT1	References
EGR1	NM_001964	early growth response 1	-2.72	Pos	-	[1]
FOS	BC004490	v-fos FBJ murine osteosarcoma viral oncogene homolog	-2.41	Pos	-	[1]
CXCL2	M57731	chemokine (C-X-C motif) ligand 2	-1.93	Pos	-	[2]
SOX9	NM_000346	SRY (sex determining region Y)-box 9	-1.72	Neg	-	[2]
THBS1	NM_003246	thrombospondin 1	-1.63	Pos	Pos	[2, 3]
ICAM1	NM_000201	intercellular adhesion molecule 1	-1.60	Pos	Pos	[2, 4]
CXCL1	NM_001511.3	chemokine (C-X-C motif) ligand 1 (melanoma growth stimulating activity, alpha)	-1.99	-	Pos	[3]
CHI3L1	NM_001276	chitinase 3-like 1 (cartilage glycoprotein-39)	1.65	Pos	-	[5]

RNA-sequence data was analyzed as stated in methods. Identification of all differentially expressed genes was based on a cutoff false detection rate fdr < 0.01, and a fold change FC ≥ 1.5. The absolute value of FC is the magnitude of up- or down-regulation for each gene/homolog after C188-9 treatment. FC > 1.5 indicates up-regulation, and < -1.5 indicates down-regulation. The genes in this table are arranged in decreasing order of FC. It also shows literature info on whether a gene is reported to be positively (Pos) or negatively (Neg) regulated by STAT3 (Column 4) and/or STAT1 (Column 5) along side the relevant reference(s).

REFERENCES

1. Song H, Jin X and Lin J. Stat3 upregulates MEK5 expression in human breast cancer cells. Oncogene. 2004; 23:8301-8309.
2. Dauer DJ, Ferraro B, Song L, Yu B, Mora L, Buettner R, Enkemann S, Jove R and Haura EB. Stat3 regulates genes common to both wound healing and cancer. Oncogene. 2005; 24:3397-3408.
3. Khodarev NN, Beckett M, Labay E, Darga T, Roizman B and Weichselbaum RR. STAT1 is overexpressed in tumors selected for radioresistance and confers protection from radiation in transduced sensitive cells. Proc Natl Acad Sci U S A. 2004; 101:1714-1719.
4. Bezerra DP, Castro FO, Alves AP, Pessoa C, Moraes MO, Silveira ER, Lima MA, Elmiro FJ and Costa-Lotufo LV. In vivo growth-inhibition of Sarcoma 180 by piplartine and piperine, two alkaloid amides from Piper. Brazilian journal of medical and biological research = Revista brasileira de pesquisas medicas e biologicas / Sociedade Brasileira de Biofisica [et al]. 2006; 39:801-807.
5. Clarkson RW, Boland MP, Kritikou EA, Lee JM, Freeman TC, Tiffen PG and Watson CJ. The genes induced by signal transducer and activators of transcription (STAT)3 and STAT5 in mammary epithelial cells define the roles of these STATs in mammary development. Mol Endocrinol. 2006; 20:675-685.
