# Supplementary material for: Small-molecule inhibition of STAT3 in radioresistant head and neck squamous cell carcinoma
Source: Oncotarget. 2016 Mar 25;7(18):26307–30. doi: 10.18632/oncotarget.8368 (PMC5041982; doi:10.18632/oncotarget.8368)
Supplement: Supplementary file 2 [file oncotarget-07-26307-s002.docx]

**Supplemental Table 1**. Summary of STAT inhibitory activities (IC_50_ ± SD, n≥ 2) of C188 and 39 other compounds obtained by 2D similarity screening and 3D pharmacophore search of chemical libraries

| **Sl** | **Compound** | **Structure** | **pY-Peptide Binding**  **(SPR)** | **Ligand Induced Phosphorylation**  **(Phosphoflow)** | | |
| --- | --- | --- | --- | --- | --- | --- |
|  |  |  | **(IC50 ± sd)** | **pSTAT3 (GCSF) (IC50 ± sd)** | **pSTAT5 (G-CSF) (IC50 ± sd)** | **pSTAT1 (IFN-γ) (IC50 ± sd)** |
| 1 | C188 | 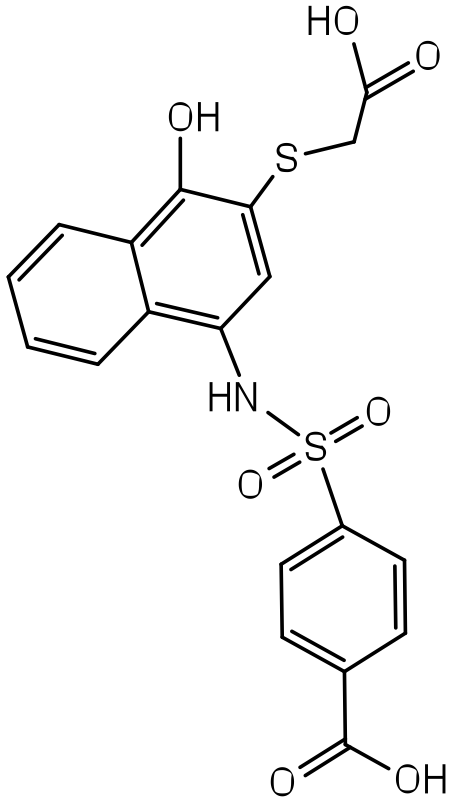 | 7.5 ± 3.5 | 16.8 ± 20.1 | 40.0 ± 0.0 | 15.0 ± 0.0 |
| 2 | C188-1 | 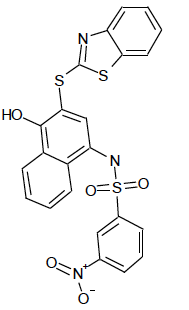 | 5.5 ± 2.1 | 16.2 ± 24.1 | 1.5 ± 0.0 | 50.5 ± 53 |
| 3 | C188-2 | 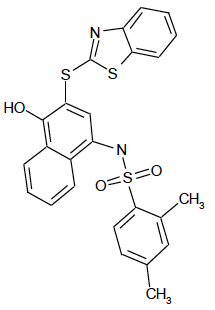 | 20.0 ± 9.9 | 29.8 ± 25.9 | 35 ± 29.7 | 51.0 ± 0.0 |
| 4 | C188-3 | 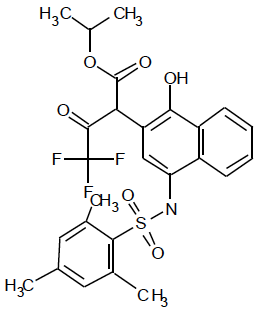 | 17.3 ± 8.5 | 21.6 ± 11.1 | 21 ± 0.0 | 34.5 ± 10.6 |
| 5 | C188-4 | 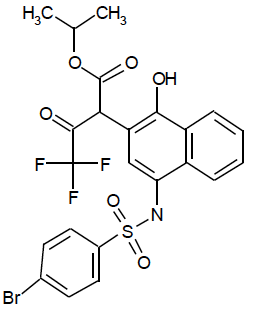 | 21.5 ± 21.9 | 11.0 ± 1.4 | 203.7 ± 166.9 | 29.0 ± 0.0 |
| 6 | C188-5 | 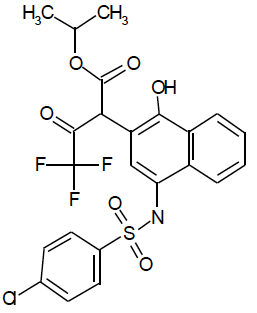 | 11.5 ± 7.8 | 27.0 ± 1.4 | NA | 163.0 ± 193.7 |
| 7 | C188-6 | 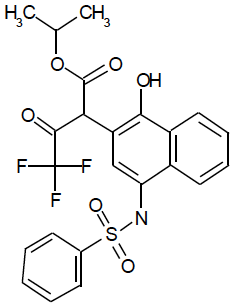 | 16 ± 15.6 | 23.5 ± 6.4 | 212.3 ± 151.8 | 132.3 ± 145.2 |
| 8 | C188-7 | 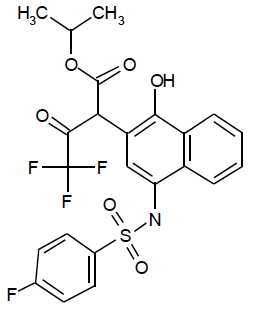 | 4.7 ± 2.1 | 10.5 ± 13.4 | NA | 35.0 ± 39.6 |
| 9 | C188-8 | 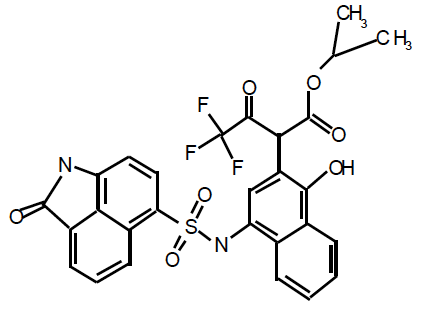 | 4.5 ± 2.1 | 3.4 ± 0.9 | 23 ± 0.0 | 35.0 ± 25.2 |
| 10 | C188-9 | 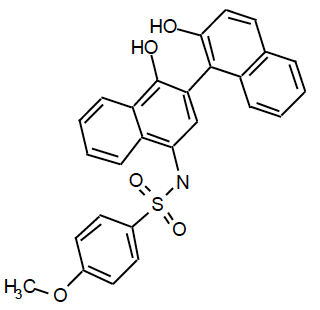 | 2.5 ± 2.1 | 8.9 ± 5.8 | 5.1 ± 3.6 | 9.6 ± 5.6 |
| 11 | C188-10 | 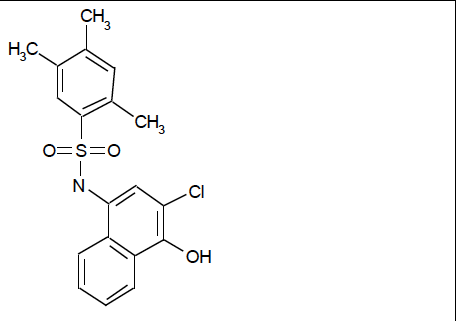 | 8.0 ± 2.8 | NA | NA | NA |
| 12 | C188-11 | 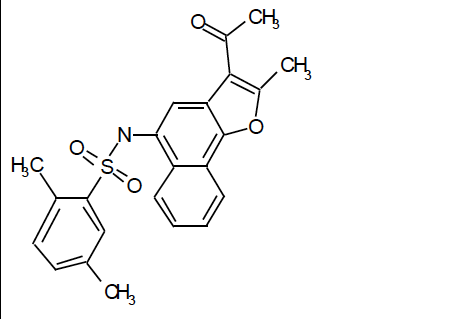 | 54.5 ± 40.3 | NA | NA | NA |
| 13 | C188-12 | 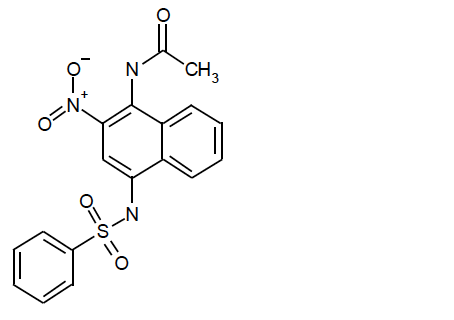 | NA | NA | NA | NA |
| 14 | C188-13 | 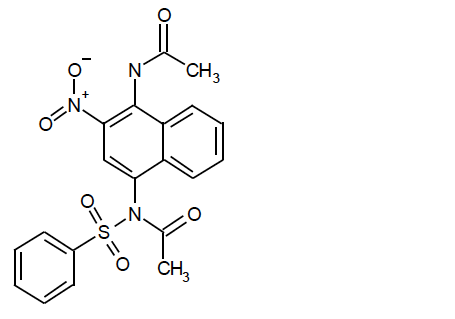 | 280.0 ± 41.0 | 155.0 ± 205.1 | NA | NA |
| 15 | C188-14 | 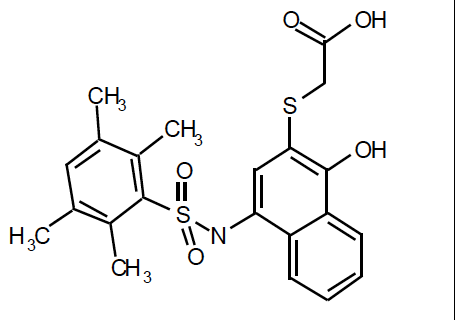 | 8.7 ± 9.1 | 40.3 ± 50.8 | 10 ± 0.0 | 57.0 ± 0.0 |
| 16 | C188-15 | 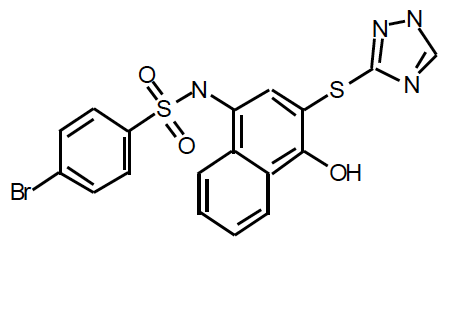 | 1.5 ± 0.7 | 3.3 ± 0.2 | 12 ± 1.4 | ND |
| 17 | C188-16 | 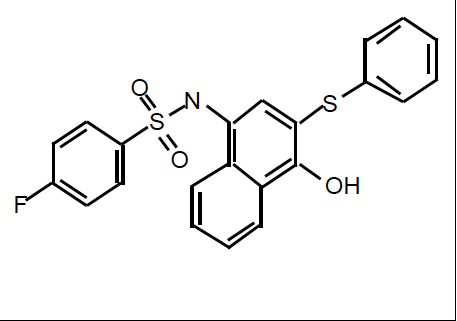 | 4.0 ± 0.0 | 169.0 ± 185.3 | NA | NA |
| 18 | C188-17 | 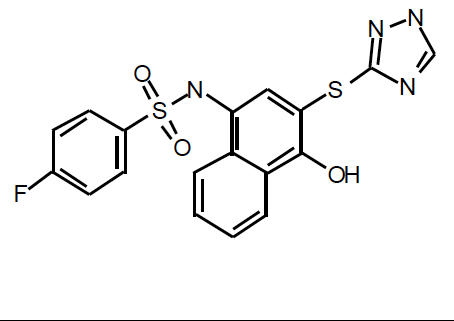 | 3.5 ± 2.1 | NA | 155 ± 205.1 | NA |
| 19 | C188-18 | 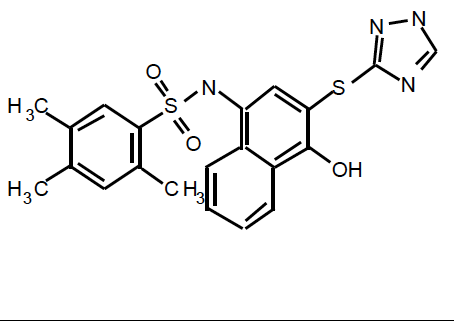 | 3.5 ± 0.7 | NA | NA | NA |
| 20 | C188-19 | 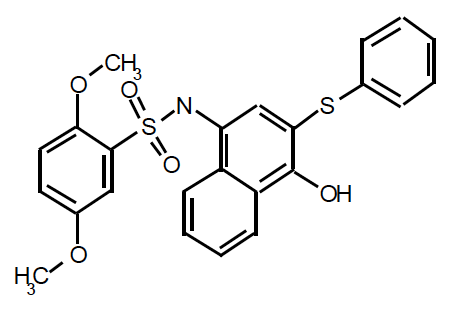 | 5.5 ± 4.9 | NA | NA | NA |
| 21 | C188-20 | 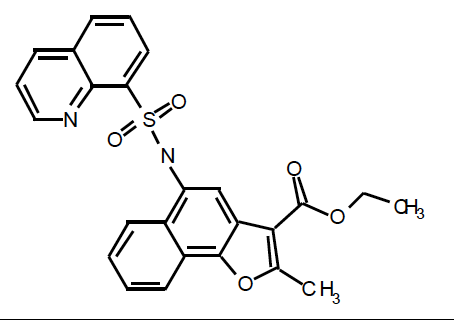 | 27.0 ± 25.5 | NA | NA | 9.0 ± 0.0 |
| 22 | C188-21 | 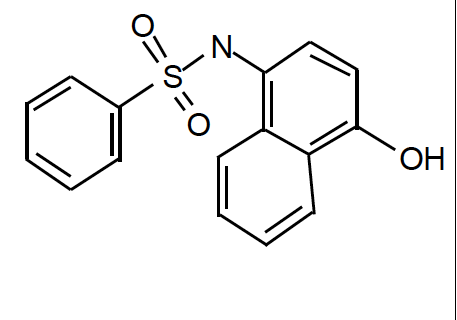 | NA | 15.0 ± 17.0 | 167.5 ± 187.4 | NA |
| 23 | C188-22 | 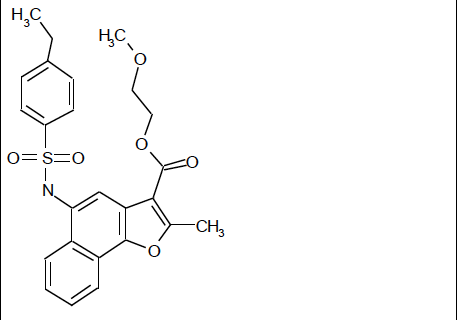 | 73.0 ± 17.0 | NA | NA | NA |
| 24 | C188-23 | 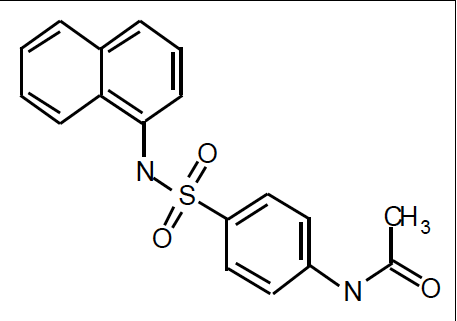 | NA | NA | NA | NA |
| 25 | C188-24 | 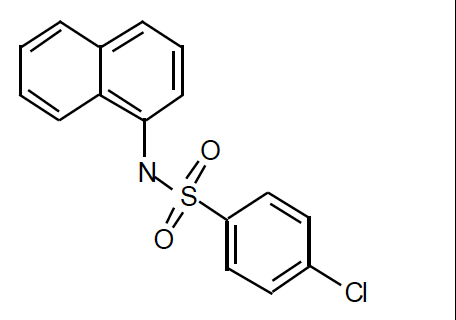 | NA | NA | NA | NA |
| 26 | C188-25 | 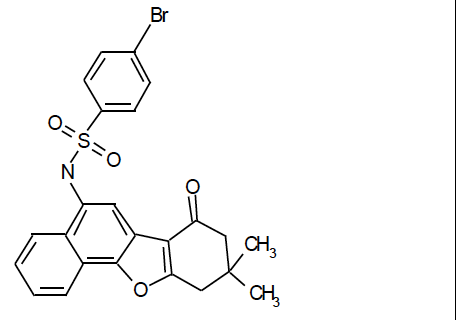  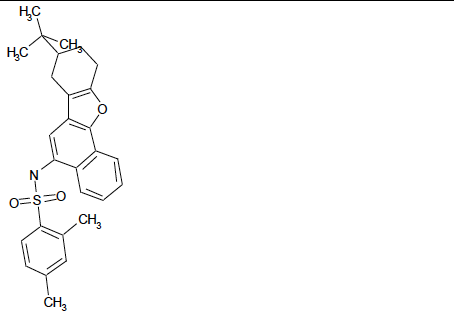 | 45.0 ± 8.5 | NA | NA | 17.0 ± 0.0 |
| 27 | C188-26 | 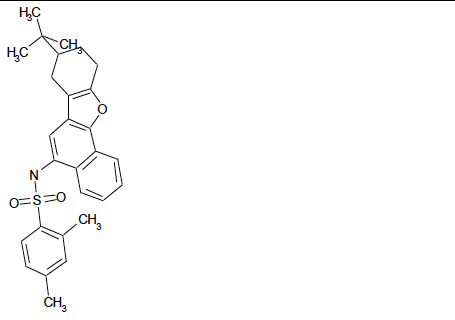 | 89.0 ± 2.8 | 196.5 ± 146.4 | NA | 155.0 ± 205.1 |
| 28 | C188-27 |  | NA | 155.0 ± 205.1 | NA | NA |
| 29 | C188-28 | 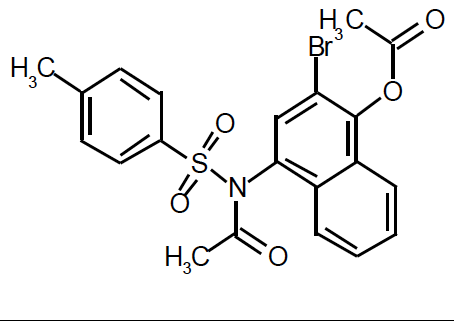 | 71.0 ± 33.9 | 213.5 ± 122.3 | NA | 10.0 ± 0.0 |
| 30 | C188-29 | 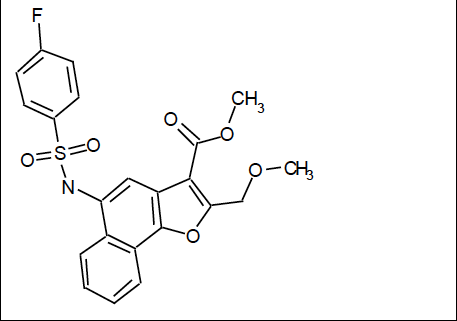 | 27.5 ± 10.6 | 214.5 ± 120.9 | NA | NA |
| 31 | C188-30 | 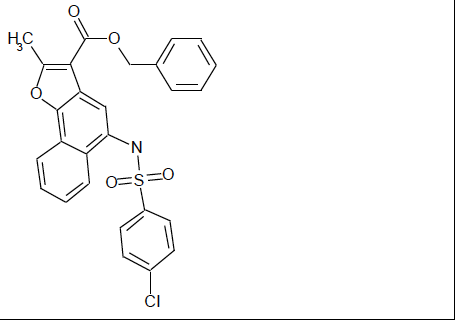 | 70.5 ± 70 | NA | NA | NA |
| 32 | C188-31 | 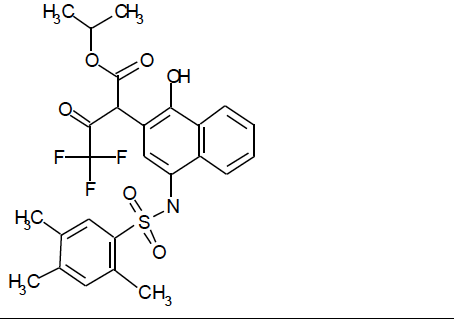 | 42.0 ± 7.1 | 13.0 ± 0.0 | NA | 47.0 ± 0.0 |
| 33 | C188-32 | 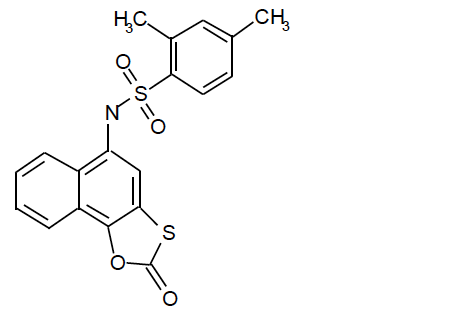 | 48.0 ± 19.8 | 7.5 ± 3.6 | NA | 3.0 ± 0.0 |
| 34 | C188-33 | 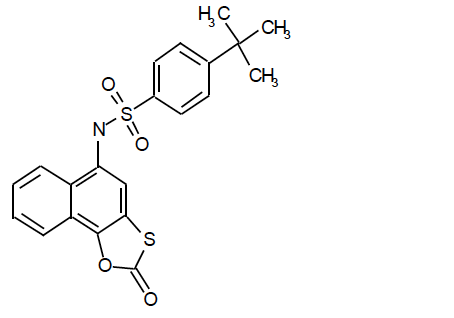 | 20.0 ± 4.2 | 7.9 ± 5.8 | 6 ± 0.0 | 10.0 ± 0.0 |
| 35 | C188-34 | 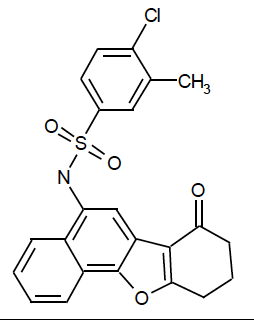 | 13.5 ± 3.5 | NA | NA | NA |
| 36 | C188-35 | 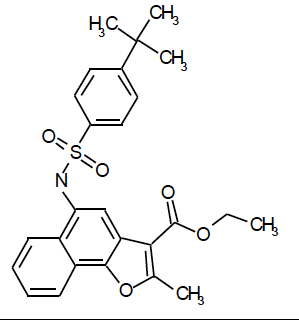 | 25.5 ± 6.4 | 219.0 ± 114.6 | NA | NA |
| 37 | C188-36 | 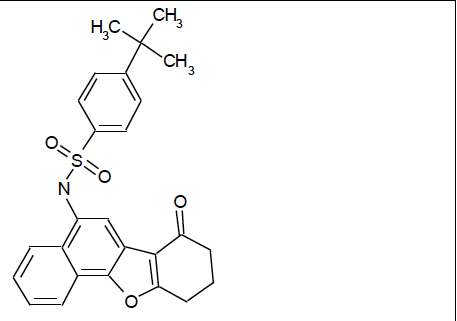 | 31.5 ± 7.8 | NA | NA | NA |
| 38 | C188-37 | 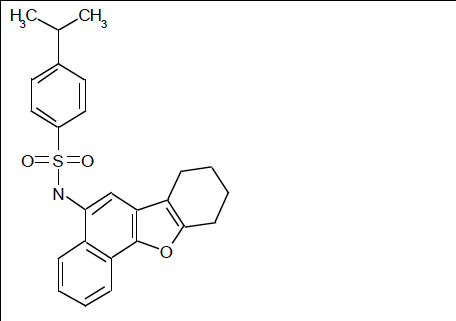 | 24.5 ± 6.4 | NA | NA | NA |
| 39 | C188-38 | 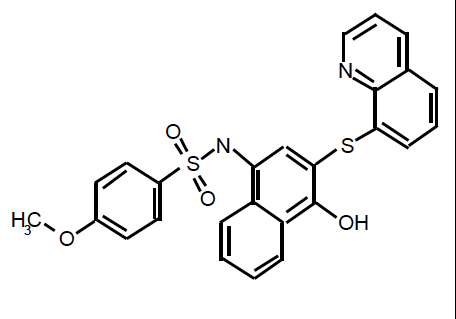 | 18.5 ± 9.2 | ND | ND | NA |
| 40 | C188-39 | 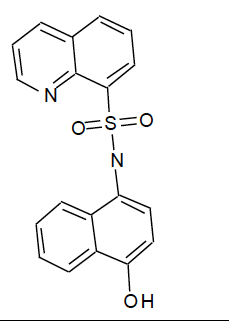 | NA | NA | NA | NA |

Data represented are IC50 values (μM), obtained using results from multiple experiments (n ≥ 2) except those having no activity (NA) or very high activity (>300 μM) in single runs; ND: Not Done; Cells used: IL6/sIL6R induced STAT3 nuclear translocation by High Throughput Fluorescent Microscopy (HTFM), STAT3-α-GFP expressing MEFs; G-CSF induced pSTAT3/5 and IFN-**γ** induced pSTAT1 by Phosphoflow: Kasumi-1
